# Characterization of a novel *SERPINA1* variant carrying two missense mutations: molecular mechanisms and functional impact

**DOI:** 10.1186/s13023-025-04142-z

**Published:** 2025-11-21

**Authors:** Celine Leon, Marie-Françoise Odou, Bertrand Roquelaure, Louis Lebreton, Mathias Ruiz, Carolin Victoria Schneider, Celine Renoux, Aurélie Evrard, Malika Balduyck, Magali Dechomet, Christine Lombard, Mathilde Butori-Pepino, Kai Markus Schneider, Victor Marin, Sylvaine di-Tomasso, Cyril Dourthe, Jean-William Dupuy, Anne-Aurélie Raymond, Sophie Collardeau-Frachon, Aurélie Haffner, Radia Fritih, Emmanuelle Goubert, Vanna Geromel, Philippe Joly, Alexandre Fabre, Marion Bouchecareilh

**Affiliations:** 1 CNRS, INSERM, BRIC, University of Bordeaux, Bordeaux, U1312 France; 2 CHU Lille, Department of Biochemistry and Molecular Biology ‘Hormonologie, Métabolisme-Nutrition, Oncologie’, Lille, France; 3 Department of Pediatrics, Hôpital de la Timone Enfants, Assistance-Publique des Hôpitaux de Marseille (AP-HM), Marseille, France; 4 Département de Biochimie, Hôpital Pellegrin, Centre Hospitalier Universitaire de Bordeaux, Bordeaux, France; 5 Department of Pediatric Hepatology, Gastroenterology and Nutrition, French Reference Center for Biliary Atresia and Genetic Cholestasis, European Reference Network Rare-Liver, Hôpital Femme-Mère-Enfant, Hospices Civils de Lyon, Lyon, France; 6 Department of Gastroenterology, Metabolic Diseases and Intensive Care, University Hospital RWTH Aachen, Aachen, Germany; 7 Service de Biochimie et de Biologie Moléculaire, Centre de Biologie et de Pathologie Est, Hospices Civils de Lyon, Bron, France; 8 CNRS, INSERM, Univ. Bordeaux, TBM-Core, US5, UAR 3427, OncoProt, Bordeaux, F-33000 France; 9 Department of Pathology, Hôpital Femme Mère Enfant, Hospices Civils de Lyon, Lyon, France; 10 Department of Pathology, Hôpital de la Timone, Assistance-Publique des Hôpitaux de Marseille (AP-HM), Marseille, France; 11 Service de génétique-oncogénétique moléculaire Eurofins-Biomnis, Lyon, France; 12 MMG, INSERM, Aix Marseille University, Marseille, France; 13 Department of Biological Immunology, Hôpital Lyon Sud, Hospices Civils de Lyon, Lyon, France; 14 Pediatric Gastroenterology Department, Hôpitaux pédiatriques de Nice CHU-Lenval, Nice, France; 15 Univ. Lille, Inserm, CHU Lille, U1286 – Infinite, Lille, F-59000 France; 16 EA 7364 RADEME, laboratoire de biochimie et biologie moléculaire, Université de Lille, Lille, France

**Keywords:** Alpha-1 antitrypsin deficiency, Genetic liver disease, Rare liver disease, New variant

## Abstract

Alpha 1-Antitrypsin Deficiency (AATD) is a rare genetic disorder caused by mutations in the *SERPINA1* gene, which encodes the Alpha 1-Antitrypsin (AAT) protein. Individuals carrying pathogenic *SERPINA1* variants are predisposed to lung and liver damage. The most common AATD-associated *SERPINA1* alleles are the S and Z alleles, though additional variants have been identified. This study describes the discovery of a new *SERPINA1* variant detected in two unrelated families from two cities in southeastern France. This variant, named PiZ_marseille_, results from *in cis* combination of the PiZ allele and a rare variant known as PiZ_bristol_. PiZ_marseille_ has been associated with early-onset liver disease in childhood. To further investigate this new variant, we performed its molecular and functional characterization, revealing that PiZ_marseille_ shares the pathogenic properties of both the PiZ and PiZ_bristol_ variants. These properties include its retention in the endoplasmic reticulum as aggregates and its degradation through the autophagy and proteasome pathways. Additionally, we conducted proteomic profiling to explore the association of this mutant with liver disease. Our analysis revealed that the neutrophil degranulation pathway is particularly deregulated in PiZ_marseille_ liver samples. Furthermore, when compared to other AAT genotypes, the proteomic profile of PiZ_marseille_ most closely resembles that of the PiZ variant, rather than other *SERPINA1* variants.

**Supplementary Information:**

The online version contains supplementary material available at 10.1186/s13023-025-04142-z.

## Introduction

Alpha 1-antitrypsin (AAT) deficiency (AATD) is an inherited disorder characterized by reduced levels of the AAT protein in the blood. This condition predisposes affected individuals to various complications, particularly those involving the liver and lungs [1]. AAT is a crucial serine protease inhibitor primarily synthesized by hepatocytes. Its main physiological role is to protect tissues from enzymatic degradation by neutrophil elastase, cathepsin G and proteinase 3, particularly in the lungs where it prevents excessive tissue damage. Consequently, AATD is associated with emphysema due to impaired inhibition of neutrophil elastase in the lungs [1, 2]. Additionally, some AAT variants can lead to liver damage [1, 3, 4]. The AAT protein, which belongs to the serpin superfamily, is encoded by the *SERPINA1* gene located on chromosome 14. AATD is caused by pathogenic variations in this gene, resulting in defective or insufficient production of functional AAT [3]. Recent advancements in genetic screening and molecular diagnosis have facilitated the identification of several variants of the AAT protein. To date, more than 150 AAT variants have been discovered [5–7]. Some of these variants can alter the structure, function or circulating levels of AAT, contributing to the variability in clinical manifestations observed in individuals with AATD [3]. Understanding the spectrum of AAT variants is essential for accurate diagnosis, prognosis, and therapeutic management of affected patients.

The biological diagnosis of AATD involves a combination of techniques [2]. The measurement of serum AAT levels is the first step in the diagnostic approach to AATD. A serum AAT concentration below the normal value (1.1 g/L) indicates a deficiency, which can be classified as mild (0.9–1.1 g/L), intermediate (0.57–0.9 g/L), or severe ( < 0.57 g/L) [2]. The identification of AAT variants is performed through phenotyping and genotyping. AAT phenotype characterization is based on protein migration patterns, typically assessed using isoelectric focusing (IEF) [2, 8]. Although this method allows for the characterization of approximately 30 normal and pathological variants, it also has some limitations [2]. Indeed, this method, which requires sufficient expertise, is unable to detect all variants. For example, some rare variants (such as M_malton_, M_palermo_, and M_heerlen_) exhibit an isoelectric point similar to that of the normal alleles, and IEF is unable to detect null (or Q_0_) alleles [9]. Genotyping using allele-specific PCR methods may also fail to identify some rare and null alleles [10, 11]. As a result, sequence analysis of the AAT gene is considered as the reference method for identifying and characterizing rare and null variants of AAT.

AAT alleles, named with the prefix ‘Pi’ (protease inhibitor) to reflect their role in inhibiting protease activity, are categorized based on the migration patterns of mutant AAT during IEF. The normal wild-type (WT) allele is designated as PiM due to its medium migration velocity. In contrast, the PiS (rs17580) and PiZ alleles (rs28929474), which are the most common variants, exhibit slow and very slow migration velocities, respectively [1].

Unlike the PiS variant, the PiZ variant is not only the most common but also the most clinically significant variant associated with AATD. It is characterized by a point mutation (NM_000295.5:c.1096 G > A; p.Glu366Lys) that causes the misfolding and polymerization of AAT within the endoplasmic reticulum (ER) of hepatocytes. This intracellular accumulation of misfolded Z-AAT aggregates triggers cellular stress responses and hepatocyte injury, predisposing PiZ allele carriers—particularly individuals homozygous for this allele (PiZZ genotype)—to liver injury, which primarily manifests as neonatal cholestasis, fibrosis, cirrhosis, and hepatocellular carcinoma in adulthood [1, 3, 4]. Additionally, the PiZ allele, whether in the homozygous or heterozygous state, confers an increased risk of emphysema due to impaired AAT secretion and elastase inhibition in the lungs. Beyond the PiZ variant, numerous rare AAT variants have been identified, each associated with unique structural alterations and clinical implications.

In this report, we describe a newly identified variant, named PiZ_marseille_, which was initially discovered in a three-generation family from Marseille, France, affected by AATD-related liver dysfunction and a few months later in another unrelated family from Nice, France. This variant is characterized by the presence of two distinct *SERPINA1* missense mutations *in cis*: the PiZ and PiZ_bristol_ variants, the latter being another pathogenic AAT variant. We subsequently performed a molecular characterization of this composite variant and conducted proteomic profiling to investigate its association with liver disease.

## Materials and methods

### Study approval

Human samples (Supplemental Table 6) were obtained from the “Tissu-Tumorothèque Est, CRB-HCL” and “AP-HM Hôpital de la Timone” according to standard protocols and written informed consent was obtained from all participants or their legal guardians included in the study. Pediatric liver control samples were obtained from patients with diagnoses of oxalosis. The study protocol conforms to the ethical guidelines of the 1975 Declaration of Helsinki as reflected in a priori approval by the institution’s human research committee. Regarding the UK Biobank (UKB), this study was approved by the North West Multi-centre Research Ethics Committee (REC reference: 16/NW/0274) under the UKB access number 71,300.

### AAT measurement and phenotyping

AAT serum level was measured by an immunoturbidimetric automated method (reference values range, 0.9–2.0 g/L). AAT immunophenotyping was carried out by IEF using the Hydragel 18 AAT Isofocusing® kit (Sebia, Diagnostic Department, Lisses, France).

### Sanger sequencing

*SERPINA1* gene was sequenced on an ABI Prism 3500 DX device (Applied Biosystems) with previously described primers [12].

### DNA constructs

Human M and Z variants were previously cloned into the pcDNA3.1(+) plasmid. The human Z_-bristol_-*SERPINA1* and Z_marseille_-*SERPINA1* variants were generated by site-directed mutagenesis on *M*-*SERPINA1* and Z-*SERPINA1* variant open reading frame respectively, using 2 internal primers containing the Z_bristol_-*SERPINA1* mutation (forward 5’ TCA TGG AGA TTC CGG AGG C 3’ and reverse 5’ G CCT CCG GAA TCT CCA TGA 3’) and 2 external primers containing the EcoRI and BamHI sequence for subsequent cloning into the pcDNA3.1(+) plasmid (forward 5’ ATC GC GAATTC ATG CCC TCG AGC GTC TCG 3’ and reverse 5’ AT TCT AGA CTA TTT TTG GGT GGG ATT CAC 3’).

### Cell culture

HEK293A (Human Embryonic Kidney) cells were cultured in DMEM (4,5 g/L glucose with glutamine and sodium pyruvate) containing 10% (v/v) fetal bovine serum (FBS). The cells were transfected with 1.6 µg of plasmid with the Lipofectamine 200 reagent (Invitrogen) following the manufacturer’s guidelines and selected with 1 000 µg/ml of geneticin (Euromedex). Cells were treated during 24 hours with 50 µmol/L Bortezomib or for 16 hours with 50 nmol/L Bafilomycin A1 (Sigma, B1793).

### AAT secretion assays

Cells were washed with PBS and incubated with FBS-free culture medium. After 3 hours incubation, media were taken and centrifuged at 1500 rpm for 10 min at 4 °C to separate cell debris and medium. The plated cells were harvested and cell lysates were prepared in lysis buffer (50 mmol/L Tris-HCl, 150 mmol/L NaCl, 1% (v/v) Triton X-100 and protease inhibitors at 2 mg/mL of lysis buffer). Protein concentrations were then determined by the Bradford protein assay (Thermo Scientific, Rockford, IL).

### Separation of soluble and insoluble AAT fractions

Cell lysates were prepared in the same lysis buffer and centrifuged at 16,000 g for 20 min at 4 °C to separate soluble and insoluble forms of AAT. The protein concentrations of the soluble fractions were determined by the Bradford protein assay (Thermo Scientific, Rockford, IL). The insoluble pellets were resuspended in 50 mmol/L Tris HCl pH 6.8, 5%SDS, 10% glycerol buffer and sonicated.

### Immunoblotting

Total proteins, media, soluble and insoluble forms were denaturated with 1% SDS sample buffer containing β-mercaptoethanol and incubated at 95 °C for 5 minutes. The insoluble forms, 15 µg of total proteins or soluble forms and 10–15 µL of media were then separated on a 10% (v/v) SDS-PAGE, transferred to nitrocellulose membrane and immunoblotted with anti-human AAT antibody (1/2500 - Immunology Consultants Laboratory, Newberg, OR). Detection was performed with the appropriate fluorescent (infrared) secondary antibody using Chemidoc instrument (Biorad). The stain free system was used as loading control (Biorad).

### Molecular modeling tools

Different softwares were used to predict the impact of the missense variations on the AAT protein conformation. Since the two variations were transmitted in *cis* on the same allele, we used the Protein Data Bank (PDB) crystal structure (number 5io1) of recombinant human PiZ AAT variant (http://www.rcsb.org/structure/5io1) and tested the impact of the additional PiZ_bristol_ variation on the protein structure [13]. PDB model 1qlp crystal structure of AAT (Thr in position 109 and Glu in position 366) was used as native structure. **ChimeraX** allowed manipulation of the global and local structures. According to the change, multiple positions of the new residue were calculated and could be visualized using a rotamer library based on the minimum local energy of torsional angles (named χ) of the side-chain structure of the new residue. **Matchmaker function** allowed to superimpose two molecules or chains according to their pairwise sequence alignment score. Using the function render by attribute, alpha carbon (Cα) pairs of each residue can be color-coded according to their root mean square deviation (Cα-RMSD in Ǻ) in order to estimate similarities between structures [14–16]**. COSMIS** (Contact Set MISsense tolerance) is a framework developed to quantify constraints of missense variations in a local 3D protein structure. A Z-score metric is calculated for each residue or “index site” within its closed spatial amino acid neighborhoods named “contact set”. By comparison with the same naturally evolute contact set, the Z-score allows a prediction for each index site from high to low tolerance to missense variation in its spatial 3D neighborhood. A color-coded secondary structure of AAT could be visualized online using **Miztli** (http://miztli.biokerden.eu/; bioRxiv 2021.09.15.460390; doi: 10.1101/2021.09.15.460390). **AlphaMissense** is a prediction tool for all amino acid substitutions in human proteome. Its score based on accurate protein structure and evolutionary conservation allows prediction by classifying missense variations from benign to pathogenic [17]. Other in silico predictors, such as Polyphen, ESMb1 or the REVEL score were also used but they did not allow to evaluate the impact of both mutations together.

### Liver histology

Liver biopsies were performed using US guided needles (18 G-23 mm; IMAX Katracore). Coloration was performed on 3.5 µm sections of formalin-fixed, paraffin-embedded specimen with hematoxylin phloxine saffron (HPS) for standard staining and periodic Acid Shift (PAS) with and without amylase (PAS diastase) for special staining. Immunohistochemistry was performed with the following antibodies: anti-Alpha 1-Antitrypsin (rabbit, polyclonal antibody, reference RB-367-R7, Epredia) and anti-cytokeratin 7 (mouse, monoclonal antibody, clone OV-TL 12/30, dilution 1/100, Sigma-Aldrich).

### NGS sequencing

#### Panel design

A set of 94 genes (Supplemental Table 4) known to be pathogenic or considered likely involved in various pediatric liver diseases or genetic jaundice was selected to form the gene panel (Version 5: from November 2020: chip 94). The design of this panel was realized via the Agilent design platform customed gene (Agilent, Santa Clara, CA, USA). DNA extraction: Patient’s DNA was extracted from circulating leukocytes previously obtained from peripheral blood and purified using a QiaSymphony Midi Kit^®^. It was then fragmented by sonication using a Covaris M220 kit (Covaris Inc., MS, Woburn, MA, USA). Libraries constitution: The Illumina^®^ libraries of the entire genome were prepared using the NEBNext^®^ DNA Library Prep preparation kit for Illumina^®^ (NEB Inc., Ipswich, MA, USA) on a Biomek Span 8 workstation (Beckman, Villepinte, France). Enrichment was achieved by targeted capture of the coding exons of the panel genes and their intronic bases (+/− 30 base pairs) by hybridization using the SureselectXT kit (Agilent, Santa Clara, CA, USA) robotized on a Biomek 4000 substation (Beckman, Villepinte, France). Deep intronic regions were not covered by this analysis. Sequencing device: sequencing was performed on a MiSeq Illumina^®^ medium flow sequencer.

### Sequencing carried out by the Eurofins-Biomnis molecular diagnostics laboratory

Genomic DNA was isolated from EDTA blood using the DNeasy Blood & Tissue Kits from QIAGEN SA. From 50 ng of DNA, indexed libraries are prepared and hybridized with biotinylated probes of Human Exome 2.0 Plus Comprehensive Exome Spike-in. The samples are prepared following the manufacturer recommendations. The pools of libraries are sequenced on the Illumina NovaSeq6000 sequencer in paired end (2x150 bp). The sequences are analyzed following the best practices recommended by the GATK Broad Institute, with two pipelines: Internal Pipeline: BWA-MEM2, GATK 4, with dedicated CNV module end SeqOne Pipeline.

### Proteomic analysis

Preparation of histological sections, laser microdissection cutting, protein extraction and formalin fixation reversal were carried out as previously described [18, 19]. The proteins were desalted and digested using the Single-pot, solid-phase-enhanced sample preparation (SP3) method [20]. NanoLC-MS/MS analysis were performed using a Vanquish Neo UHPLC System (Thermo Scientific) associated to Orbitrap Exploris™ 480. The peptide extracts were loaded onto a 5 mm × 300 µm ID PepMap Neo Trap Cartridge (C18, 5 µm particle size, 100 Å pore size, Thermo Scientific) and separated on an analytical column (25 cm × 75 μm ID, 1.7 µm, C18 beads, Ionopticks) at a flow rate of 300 nL/min at 50 °C using a multistep gradient of 3–25% mobile phase B (80% MeCN in 0.1% formic acid) for 45 minutes and 25–35% B for 15 minutes, 35–95% B for 1 minute and an 11-minute wash at 99% B. The mass spectrometer operated in positive ion mode at a 1.4 kV needle voltage, and data were acquired using Xcalibur 4.5 software in a data-dependent mode. MS scans (m/z 375–1500) were recorded at a resolution of *R* = 120000 (@ m/z 200), a standard AGC target, and an injection time in automatic mode, followed by a top speed duty cycle of up to 1 second for MS/MS acquisition. Precursor ions (2–6 charge states) were isolated in the quadrupole with a mass window of 2 Th and fragmented with HCD @ 30% normalized collision energy. MS/MS data were acquired with a resolution of *R* = 15000 (@m/z 200), a standard AGC target, and a maximum injection time in automatic mode. Selected precursors were excluded for 45 seconds.

Protein identification and label-free quantification (LFQ) were done in Proteome Discoverer 3.1. The CHIMERYS node using the prediction model inferys_3.0.0 fragmentation was used to identify proteins in batch mode by searching against the UniProt Homo sapiens database (82415 entries, released January 2024). Two missed enzyme cleavages were allowed for trypsin. Peptide lengths of 7–30 amino acids, a maximum of 3 modifications, charges of 2–4, and 20 ppm for fragment mass tolerance were set. Oxidation (M) and carbamidomethyl (C) were respectively searched as dynamic and static modifications by the CHIMERYS software. Only “high confidence” peptides were retained corresponding to a 1% false discovery rate at the peptide level. Minora feature detector node (LFQ) was used along with the feature mapper and precursor ions quantifier. The normalization parameters were selected as follows [1]: Unique peptides [2], Precursor abundance based on intensity [3], Normalization mode: total peptide amount [4], Protein abundance calculation: summed abundances [5], Protein ratio calculation: pairwise ratio based and [6] Missing values were replaced with random values sampled from the lower 5% of detected values. The mass spectrometry proteomics data have been deposited to the ProteomeXchange Consortium via the PRIDE [21] partner repository with the dataset identifier PXD060960.

### Mass spectrometry data processing

The quantitative data were analyzed using the free R software. Proteins identified by mass spectrometry with less than 2 specific peptides were excluded. For each patient, three technical liver tissue replicates (from serial sections) were performed. A ratio from technical replicate medians was calculated for each protein. The Wilcoxon-Mann-Whitney U-test was performed using the R package in order to specifically identify the proteins with significantly different expression (*p* < 0.05) in each group, thus corresponding to specific proteomic patterns.

### Integrative biological analyses

Pathway analyses were performed using the Ingenuity Pathway Analysis (IPA) (Qiagen) database. The ten most significantly enriched canonical pathways were selected from each genotype and compared. The functional annotations were extracted from the Gene Ontology (biological functions) and Reactome databases using the GSEA (Gene Set Enrichment Analysis) web tool (https://www.gsea-msigdb.org/gsea/index.jsp).

### UKBiobank analysis

Single nucleotide polymorphisms (SNPs) were extracted from the UK Biobank (UKB) SNP genotyping data to identify carriers of the PiZ_bristolbristol_ variant (rs199422213_A), as well as individuals harboring both the PiS and PiZ variants. Basline characteristic were compared using a t-test.

## Results

### Clinical cases presentation

**Fig. 1:**
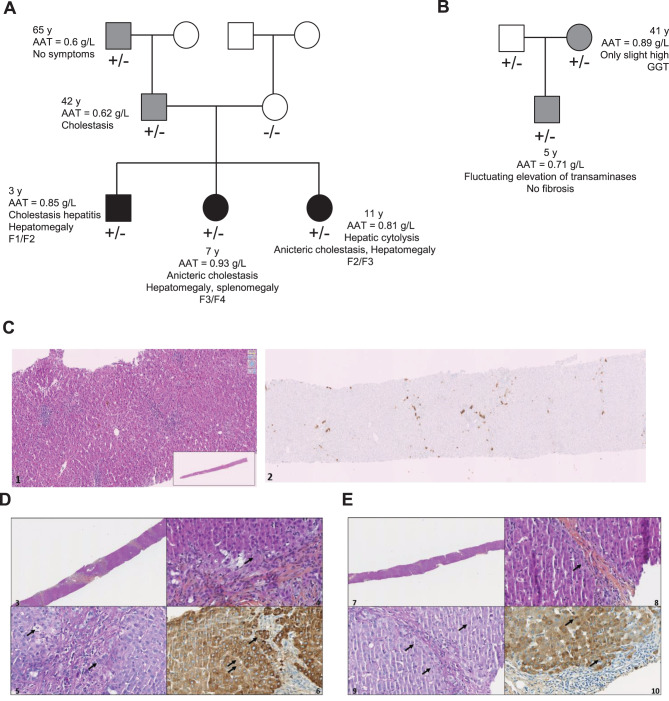
PiZ_marseille_ liver disease phenotype. **A**, **B**. Pedigree analysis chart of the Marseille and Nice family. The ± symbol indicates heterozygosity for the PiZ_marseille_ allele (haplotype: c.1096 G > A and c.326C > T). *Symptomatic patients are represented by black-filled squares and circles, whereas mild patients carrying the PiZ*_*marseille*_*variant are represented by grey-filled squares and circles.***C**. left, HPS ×10: Cholestatic liver disease with ductal plugs (centrolobular); right, CK7 ×2.5: ductopenia (best visualized by CK7 ≈ 60%) + visualized bile ducts with dystrophic appearance (clarification, lumen not clearly visible). **D**. Upper left, HPS x2.5: hepatic tissue lobulated by fibrous bridges, with discreet inflammation but without steatosis or significant ductular reaction; upper right, HPS x40: note intracytoplasmic eosinophilic globules (arrow); Lower left, pas diastase x40 : note intracytoplasmic eosinophilic globules (arrow); lower right, AAT: note intracytoplasmics globules (arrow). **E**. Upper left, HPS x2.5: hepatic tissue lobulated by thin fibrous bridges, with ductopenia and ductular reaction, without steatosis; upper right, HPS x40 : note intracytoplasmic eosinophilic globules (arrow); lower left, pas diastase x40 : note intracytoplasmic eosinophilic globules (arrow). Lower right, AAT: note intracytoplasmics globules (arrow)

The new variant was discovered a few months apart in two unrelated families from Marseille and Nice (southeastern France) who consulted specialists because their children were affected by liver dysfunction (Fig. 1A–B).

Regarding the family from Marseille, this French non-consanguineous family included three affected children: Case 1, a boy, and his two older sisters (Cases 2 and 3), as well as the father (Case 4) and paternal grandfather (Case 5) (Fig. 1A). It is to note that the mother is not a variant carrier and did not present any respiratory cough on examination with normal liver function tests.

**Case 1** is a 3-year-old boy, born at term via vaginal delivery at 40 weeks + 6 days. Due to intestinal hyperechogenicity during the second trimester of pregnancy, the parents were tested for cystic fibrosis mutations, with negative results. Neonatal adaptation was good but cholestatic jaundice without stool discoloration and hepatomegaly were discovered during the first month of life, along with poor weight gain from birth due to feeding and swallowing difficulties. Biological tests revealed disturbed liver function with cholestatic hepatitis and AAT level at 0.85 g/L (Supplemental Table 1 and Figure S1). Liver ultrasound showed no dilated bile ducts or evidence of hepatopathy or portal hypertension but revealed hyper echogenicity of the periportal spaces. At 2 months of age, hepatic biopsy revealed intrahepatic cholestasis secondary to bile duct alterations with 60% ductopenia (Fig. 1C) along with mild portal fibrosis (F1/F2) and portal/lobular inflammation, predominantly lymphohistiocytic with eosinophils. According to the age of this patient, no intracytoplasmic periodic acid–Schiff (PAS)-*positive*, *diastase*-resistant (PAS+) globules, a histologic hallmark of AATD were observed. Over the following weeks, cholestasis parameters and liver function tests did not improve (Supplemental Figure S1). Further testing excluded other causes of neonatal cholestasis, such as congenital infections and metabolic diseases (Supplemental Table 2). At 2 months of age, weight gain improved slowly with additional nasogastric tube feeding, maintained for 4 months.

**Case 2** is a 7-year-old girl, born at term with a birth weight of 3240 g, following an uneventful pregnancy. Hepatomegaly, hepatalgia and splenomegaly were discovered at 5.5 years of age during a consultation for her brother. She presented with anicteric cholestasis and an AAT concentration of 0.92 g/L. There was no additional symptom but upper endoscopy revealed grade I-II esophageal varices. Abdominal ultrasound showed signs of hepatopathy including heterogeneous liver parenchyma, incipient dysmorphia and increased liver stiffness (12 kPa). The ultrasound also revealed homogeneous splenomegaly and signs of portal hypertension such as repermeabilization of the umbilical vein. The elastogram showed increased splenic stiffness (33 kPa). Hepatic histology revealed liver parenchymal heterogeneity with fibrosing hepatopathy (AI F3/F4 METAVIR score), acute cholangiolitis, biliary duct dystrophy, ductular neogenesis without ductopenia, and intracytoplasmic AAT globules, without steatosis or significant inflammation (Fig. 1D).

**Case 3** is an 11-year-old girl, born at term with a birth weight of 3850 g, following an uneventful pregnancy. Systematic screening prompted by the siblings’ diseases revealed hepatomegaly, with no splenomegaly or signs of portal hypertension. As her sibling (case 2), she exhibited hepatic cytolysis with anicteric cholestasis and an AAT level of 0.81 g/L (Supplemental Table 1). Abdominal ultrasound showed normal liver contours and echostructure, with a regular liver capsule and still no signs of portal hypertension. Hepatic elastography was 7.4 kPa, while splenic elastography was normal at 12.7 kPa. A hepatic biopsy revealed cholestatic hepatopathy with fibrosis (AI F2/F3 METAVIR score), acute cholangiolitis, ductopenia with ductular neogenesis, and intracytoplasmic AAT globules, but without steatosis or significant inflammation (Fig. 1E). For cases 2 and 3, no hepatotropic viruses were identified on liver biopsy or plasma, nor was there any evidence of *Coxiella burnetii* infection. All three children were being treated with ursodesoxycholic acid. Biological liver test results have remained relatively unchanged to date with a slight decrease in Gamma-Glutamyl transpeptidase (GGT) levels (Supplemental Table 1).

**Case 4**, the father, is a 42-year-old man with neither significant medical history nor associated respiratory pathology. Despite an elevated Body Mass Index (BMI) at 28.4, he has no evidence of hypercholesterolemia, blood sugar disorders or hypertension. Investigations revealed abnormal liver tests with elevated GGT levels, mildly elevated Alanine Aminotransferase (ALAT) levels, and an AAT level of 0.61 g/L, associated with isolated steatosis and fibrosis stage F0/F1 (Supplemental Table 1). Lung function tests and pulmonary CT scans were normal. Case 5, the paternal grandfather, is a 65-year-old man with no significant medical history or associated respiratory pathology, despite an AAT concentration of 0.6 g/L. He has fluctuating elevations in liver tests but no evidence of hypercholesterolemia or hypertension (Supplemental Table 1). As observed in case 4, lung function tests and pulmonary CT scans were normal.

Regarding the family from Nice, this French non-consanguineous family, not related to the Marseille family, included only one affected child (Fig. 1B). Case 6 is a 5-year-old boy with an AAT level of 0.71 g/L. The diagnosis was made based on mild chronic and fluctuating elevation of transaminases, without cholestasis, liver failure, or portal hypertension. Ultrasound showed a normal liver, with elastography measured at 5.4 kPa, and no other abnormalities. Other potential diagnoses of liver biological disturbances were ruled out. Case 7 is a 41-year-old woman, with an AAT level of 0.89 g/L and subnormal liver biology, with only GGT at the upper limit of normal. Abdominal ultrasound and elastography were normal and she had no respiratory symptoms.

### AAT phenotyping and genotyping

**Fig. 2:**
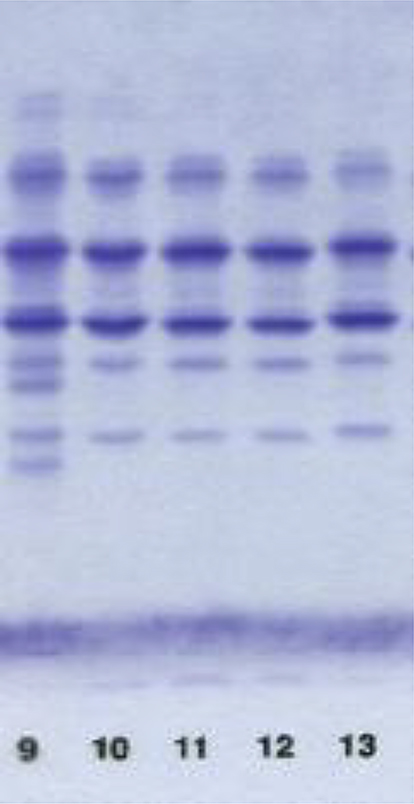
PiZ_marseille_ phenotype. Isoelectric focusing electrophoresis was used to analyze aat in plasma samples from all study subjects. Lane 9: control PiMZ, lane 10 -12: case 1–3 (siblings); 13: case 4 (father)

Due to the reduced circulating AAT concentrations (below the normal value of 1.1 g/L) in all family members, a complete AAT biological investigation was performed. It began by an analysis of AAT phenotyping by IEF on plasma samples from all subjects, which revealed a PiM profile (Fig. 2). Based on this result, we initially suspected the presence of a Null Q_0_ AAT variant or a deficient PiM-like allele (e.g., PiM_malton_). However, the direct Sanger sequencing of all four coding exons of the *SERPINA1* gene revealed, for all 5 members of the family from Marseille, the presence of two different missense variants at the heterozygous state: the first one was the well-known PiZ variant (NM_000295.5:c.1096 G > A; p.(Glu366Lys)) while the second one was identified as the PiZ_bristol_ variant (NM_000295.5:c.326C > T; p.(Thr109Met)). Given the familial segregation (three consecutive generations) of these two variants, the only plausible explanation was a *cis* transmission on the same *SERPINA1* allele, thus forming a composite new AAT variant that we decided to name PiZ_marseille_.

Interestingly, a similar scenario was observed a few months later in the second family from Nice. The biochemical AAT analysis of the affected boy revealed a PiM phenotype on IEF. Once again, direct Sanger sequencing identified both c.326C > T and c.1096 G > A variants at heterozygous state on the *SERPINA1* gene. The parental segregation study confirmed the presence of the PiZ_marseille_ allele, this time transmitted by the mother.

We therefore questioned whether the PiZ_marseille_ variant might be more common than previously anticipated. To explore this hypothesis, we analyzed exome data from UK Biobank (UKB) participants, focusing on the *SERPINA1* gene. The UKB is a population-based cohort study conducted in the United Kingdom that recruited 502,511 volunteers aged 37–73 years at baseline. Our analysis did not identify any carriers of the PiZ_marseille_ variant, suggesting an extremely low prevalence and a possible geographical restriction to France or Southern Europe. We then screened for the PiZ_bristol_ variant by Sanger sequencing in 17 PiMZ patients previously genotyped at the Bordeaux Hospital laboratory. None of these patients carried the PiZ_bristol_ variant, further confirming the extremely low prevalence of PiZ_marseille_.

Further analysis of the UKB identified 46 carriers of the PiZ_bristol_ variant and < 5 carriers of both PiS and PiZ_bristol_ variants (Supplemental Table 3). Phenotypic comparisons revealed only a significantly lower level of lipoprotein A in PiZ_bristol_ variant carriers compared to non-carriers. No significant hepatic phenotype was observed in PiS heterozygotes or PiSZ_bristol_ compound heterozygotes, suggesting that PiZ_bristol_ alone is insufficient to induce liver injury in heterozygotes.

### PiZ_marseille_ and its association with liver damage: NGS analysis

To investigate the liver damage observed in all affected siblings (cases 1–3) from Marseille, exome sequencing was performed on their entire family. This began with a cholestasis gene panel targeting genes associated with isolated and syndromic forms of cholestasis (Supplemental Table 4) [22], followed by whole-exome sequencing. Apart from variations in the *SERPINA1* gene, no potentially pathogenic variation was identified in genes known to be associated with liver disease. However, several rare variants inherited from the mother and shared by the three affected children, which may partially explain the liver injury observed, were identified (Supplemental Table 5). The impact of these variants has been assessed using in silico prediction tools: the REVEL score for single nucleotide variants (SNVs) [23] and Splice-AI for splice variants [24]. Based on the analysis, the variants *VNN1* (c.956A > G), *HGD* (c.767C > A) and *DPAGT1 (*c.626A > C) were identified as potentially damaging (Revel score exceeding 0.5) (Supplemental Table 5).

### PiZ_marseille_ molecular characterization

**Fig. 3:**
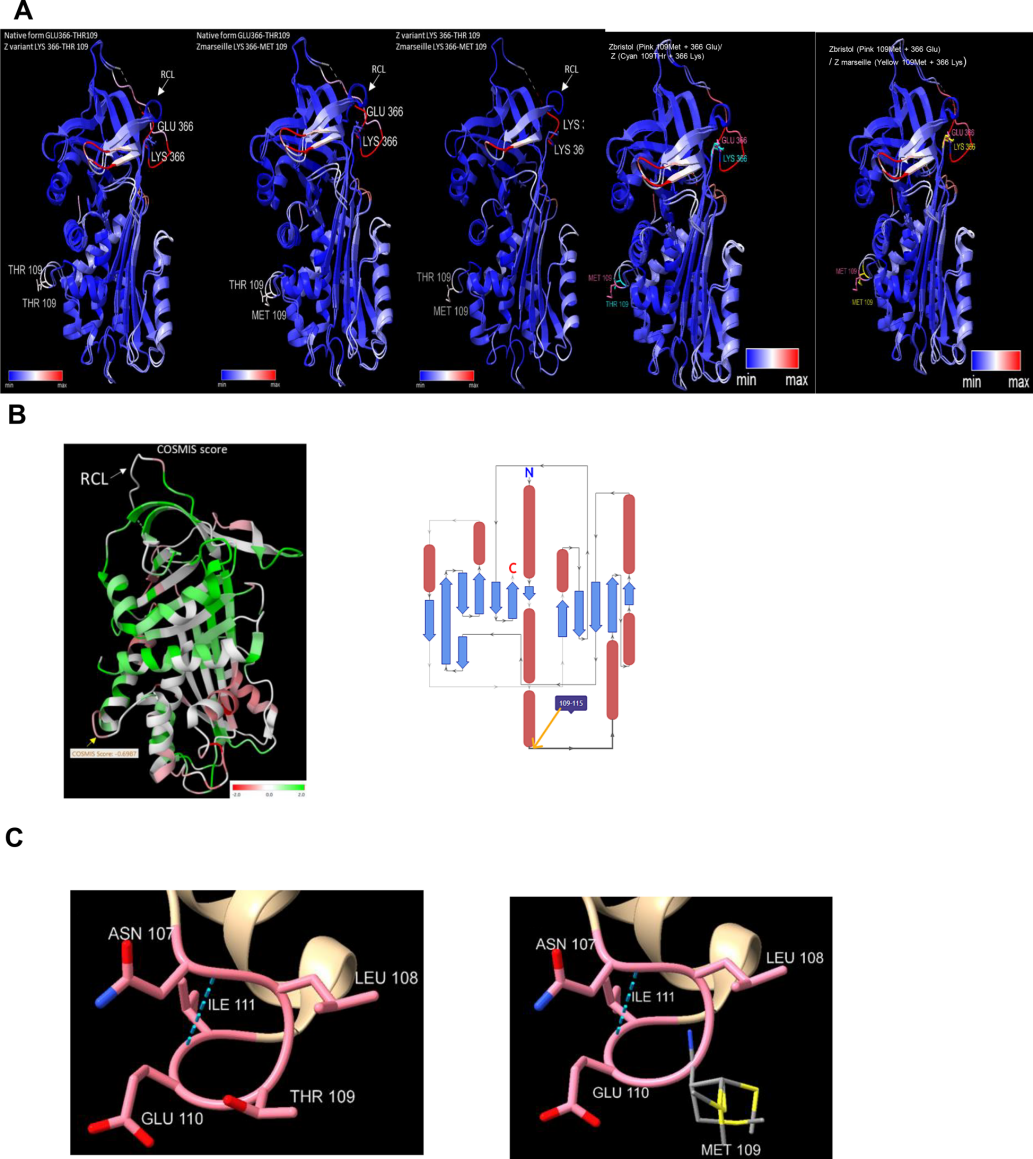
Structural modeling of the PiZ_marseille_. **A**. Superposition of aat 3D structures by pairs. From left to right: PiM vs PiZ, PiM vs PiZ_marseille,_ PiZ vs PiZ_marseille_, PiZ_bristol_ vs PiZ and PiZ_bristol_ vs PiZ_marseille_, PiZ_bristol_. RCL loop is indicated at the top, all molecules are shown with a vertical 45 degres right turn. Each pair of molecules is color-coded according to the distance between their Cα-RMSD in Ǻ. The distance varies from 0 (blue) to 2 Ǻ (white) and 4 Ǻ (red). Backbone structure of the 3 molecules is well conserved (blue color) except at site 109 and 366 due to amino-acid variations and most of the outwards segments. RCL loop appears in pink redish color at the back of the molecule due to its dynamic positions as well as the 233–239 loop generally presented at the back. Both molecules Z (5io1) and PiZ_marseille_ are perfectly superposed because PiZ_marseille_ was build using the rotamer function from ChimeraX. **B**. PiZ variant (5io1 model) color-coded according to Cosmis predictor score (left). RCL is indicated at the top of the figure (white arrow) and Thr109 (PiZ_bristol_) at the bottom left (yellow arrow), score ladder is at the bottom right. Secondary structure topology of aat (right) (from Mitzli, source PDBe and PDBsum). Yellow arrow points on the position 109 involved in PiZ_bristol_ and PiZ_marseille_. **C**. Figure at the left shows the local 3D conformation between the asn residue (codon 107) and the thr residue (codon 109) which is replaced by the four most probable positions of a Met residue in the PiZ_marseille_ variant (right), leading to steric hindrance. Asn 107 residue should not be glycosylated anymore. Hydrogen bond ile 111-asn 107 (left and right), probably implicated in the loop, is shown in turquoise blue dashed line. Amino function of Met 109 (blue-right) is shown inward in the loop and is not aligned with the amino group of thr 109 shown in pink implying slight modification of the loop itself. Computing structural effects in miztli show an additional hydrogen bound predicted between Met 109 (N atom in blue) and asn 107 (O atom- red) which might, in this case, disturb the 3D conformation of the loop. Rotamer function predicts that the four most probable orientations of Cβ atoms of the Met 109 side chain are turned outwards (right figure-atom S in yellow). Thr to Met amino-acid change also increases hydrophobicity of this position

To investigate further and determine how the PiZ_marseille_ variant might contribute to hepatocyte toxicity, we first employed molecular modeling tools. A global comparison between native AAT (PDB model 1qpl), PiZ variant (PDB model 5io1), PiZ_bristol_ and PiZ_marseille_ (PDB model 5io1 modified using the rotamer function in ChimeraX) revealed no backbone conformation difference (Fig. 3A). However, Cosmis predictor evaluated the PiZ_bristol_ variation at position 109 (Thr to Met) with a score compatible with a likely damaging or pathogenic effect (Cosmis score: −0.6987) (Fig. 3Bleft). Prediction of the secondary structure topology with Mitzli shows that position 109 in the AAT protein is at the junction between two domains of the protein (Fig. 3Bright). Changes at this position might change the environment interactions and structural folding. Constraints from this position might be important for the folding of the protein.

Local structural analysis shows an outward loop at position 109, which closely interacts with Asparagine 107, a well-known glycosylation site that might be directly affected. Thus, the change from Threonine to Methionine at position 109 is predicted to result in side chains pointing outward, increased hydrophobicity and an additional hydrogen bond, all of which might have a local structural effect (Fig. 3C).

We attempted to evaluate the impact of the association of the two variations in PiZ_marseille_. First of all, calculation of the distance between alpha-carbons of Lys366 and Met109 using ChimeraX indicates 45.380 Å, taking into account that, regarding the global 3D structure of the protein, both residues are respectively pointing forward and backward. According to this distance, it is unlikely that both domains can interact together. The right part of Fig. 3A shows the structural superimposition of PiZ_bristol_ versus PiZ_marseille_. Except the structural difference of position 366, the rest of the protein is perfectly superimposable. Therefore, we can assume that the association of the two variations is only additive and not synergistic.

**Fig. 4:**
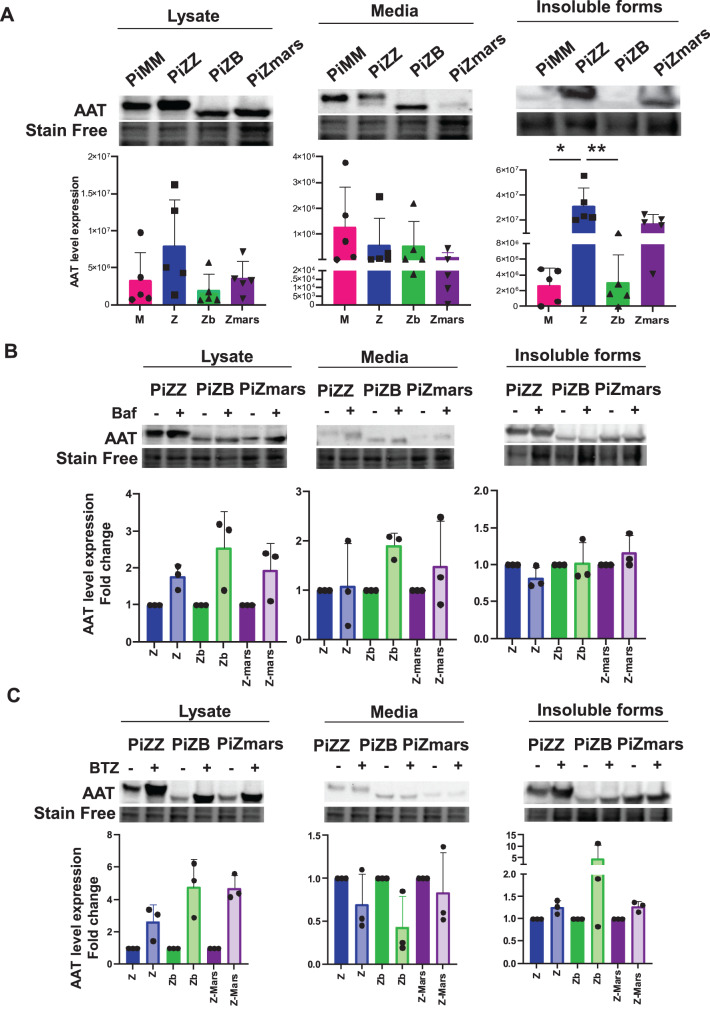
PiZ_marseille_ biogenesis characterization. **A**. immunoblot analysis of AAT protein expression from cell lysates (lysates), culture media and insoluble fractions of HEK293A cell lines expressing PiMM, PiZZ, PiZ_bristol_ (ZB) or PiZ_marseille_ (Zmars). Quantitative analysis after normalization with stain free staining are showed for 5 replicates (Kruskall Wallis test, * *p* value < 0,05 **p value < 0,01). **B**-**C**. Immunoblot analysis of AAT protein expression from cell lysates (lysates), culture media and insoluble fractions of HEK293A cell lines expressing PiMM, PiZZ, PiZ_bristol_ (ZB) or PiZ_marseille_ (Zmars) upon 16 h with 50 nM Bafilomycin (Baf-**B**) or 24 H with 50 µM Bortezomib (BTZ-**C**) treatment. Quantitative analysis after normalization with stain free staining are showed for 3 replicates (Wilcoxon test, * *p* value < 0,05 **p value < 0,01)

Next, we performed cell biology experiments using Human Embryonic Kidney (HEK293A) cell lines expressing, at the homozygous state, the human PiM, PiZ, PiZ_bristol_ or PiZ_marseille_ variants. These cell lines were generated to observe and compare the trafficking and aggregation properties of the different variants. Briefly, the trafficking of the AAT glycoprotein through the secretory pathway can be monitored by observing changes in its migration on SDS-PAGE since the normal oligosaccharide processing during ER and Golgi trafficking produces a mature slower migrating glycoform that is secreted into the extracellular medium. As expected, HEK293A cells expressing the PiMM genotype showed normal maturation, trafficking and constitutive secretion of AAT while the PiZZ genotype showed retention of aggregate forms within cells and a very low secretion (Fig. 4A). Regarding the homozygous PiZ_Bristol_ and PiZ_marseille_ variants, they both exhibited increased mobility on SDS-PAGE compared to PiMM and PiZZ variants while their intracellular levels were similar. However, the secreted levels of the PiZ and PiZ_bristol_ variants were reduced compared to the wild-type, while the PiZ_marseille_ variant was not secreted at all (Fig. 4A) suggesting it is retained and/or efficiently degraded by intracellular mechanisms. Additionally, and similarly to the PiZ variant, the PiZ_Marseille_ variant also forms aggregates, albeit to a lesser extent (Fig. 4A). Overall, these results indicate that the PiZ_marseille_ variant exhibits all the major pathogenic properties of both the PiZ_bristol_ and PiZ variants and thus appears to be a combination of them, at least at the cellular level.

To further characterize this variant, we inhibited the autophagy pathway using Bafilomycin A1 and the proteasome using Bortezomib (Fig. 4B–C). We observed that both autophagy inhibition and proteasome inhibition led to an increase in the intracellular and secreted forms of PiZ_marseille_ variant (Fig. 4B). However, the fold change was greater upon proteasome inhibition compared to autophagy inhibition (4.7 vs. 1.5), suggesting that this variant is degraded by both pathways but predominantly by the proteasome (Fig. 4B–C). Similar results were observed for the PiZ_bristol_ variant (Fig. 4B–C). All full-size images have been included in the supplementary data file. In conclusion, these results suggest that PiZ_marseille_ is partially retained as aggregates and is effectively degraded by both the autophagy and proteasome pathways.

### PiZ_marseille_ proteomic analysis

**Fig. 5:**
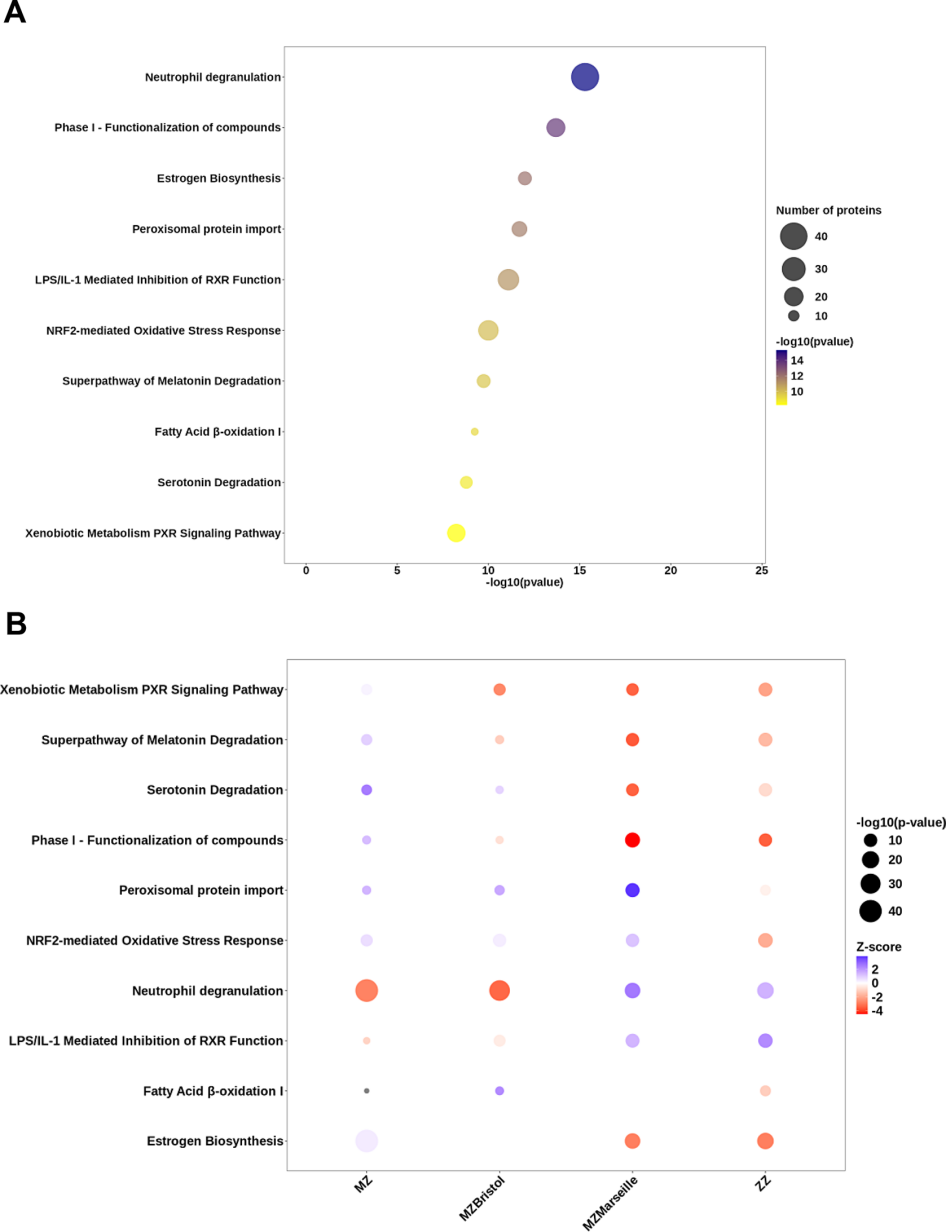
PiZ_marseille_ proteomic profiling. Biological pathways associated with (**A**) PiMZ_marseille_ or (**B**) PiZZ, PiMZ and PiMZ_bristol_ genotypes, identified by mass spectrometry analysis of FFPE liver samples. Case 1 corresponds to PiMZ_marseille_. GSEA was carried out on the Ingenuity Pathway Analysis (IPA) database (Canonical Pathways). The most significantly enriched pathways are represented for PiMZ_marseille_ genotype and compared to other AATD genotypes. Colors and circle size correspond to level of significance. Abbreviations: LXR, liver X receptor; NRF2, nuclear factor-erythroid 2-related factor 2; lps: endotoxin lipopolysaccharide; RXR, retinoid X receptors

Alongside the molecular characterization of this new variant, we investigated further the biological pathways underlying these features and performed in situ proteomic analysis. This involved combining laser microdissection of hepatocytes with mass spectrometry on human formalin-fixed paraffin-embedded (FFPE) liver tissues [18] from PiMM and PiMZ_marseille_ patients (Supplemental Table 6). We compared the relative protein abundances between PiMM hepatocytes and PiMZ_marseille_ hepatocytes (Fig. 5A) and carried out a Gene Set Enrichment Analysis (GSEA). The most significantly enriched pathway for the PiMZ_marseille_ genotype was related to neutrophil degranulation.

We then performed the same analysis for PiMZ, PiZZ, and PiMZ_bristol_ patients (Supplemental Table 6) and investigated the biological functions associated with each specific genotype (Fig. 5B). This global analysis revealed that most of the functional deregulations were shared between the PiZZ and PiMZ_marseille_ genotypes with the most significantly enriched pathway for these two genotypes being related to estrogen biosynthesis. Interestingly, following our functional study, we observed that PiZ_marseille_ variant retains all major pathogenic properties of both PiZ_bristol_ and PiZ variants (Fig. 4). However, regarding biological functions, this new AATD variant shows more similarities with PiZ variant than with PiZ_bristol_ and further supports the pathogenicity of PiZ_marseille_ variant in relation to AATD-associated liver damage.

## Discussion

In this study, the collaborative efforts of clinicians, geneticists, biologists and basic researchers led to the identification and cellular characterization of PiZ_marseille_, a novel variant of the *SERPINA1* gene causing liver damage at the heterozygous state. This discovery was made through the investigation of a family of five individuals with AATD, three of whom presenting with severe liver damage. An in-depth analysis of this family revealed that the only common feature among these cases was the presence of the PiZ_marseille_ haplotype. This latter is characterized by two heterozygous missense mutations *in cis* on a same *SERPINA1* allele: the PiZ c.1096 G > A and PiZ_bristol_ c.326C > T variants. No other genetic, radiological, metabolic or infectious factor was identified to account for the observed liver disorders. To the best of our knowledge, this study thus reports for the first time the combination of the rare PiZ_bristol_ variant *in cis* with the PiZ variant and expands the list of pathogenic *SERPINA1* variants with a PiZ genetic background. Interestingly, the PiZ_marseille_ variant was identified twice in the same year in a second independent family from the South of France. Moreover, we observed that the PiZ_marseille_ allele shares a common “M1Ala” genetic background in both families in our study. The M1Ala genetic background of the SERPINA1 gene corresponds to the presence of the c.710T > C polymorphism, which encodes a p.(Val237Ala) amino acid variation. Interestingly, the classic PiZ variant is also most often present on an “M1Ala” genetic background, suggesting that a c.326C > T gene conversion (PiZ_bristol_ variant) occurred on a PiZ allele to give rise to the PiZ_marseille_ allele, and not the reverse. Although we cannot exclude the possibility that the same gene conversion occurred independently twice on separate PiZ alleles, the most plausible explanation, given the rarity of the PiZ_bristol_ variant (allelic frequency below 3.5 × 10^−5^ in the GnomAD database), is that it occurred once and was subsequently dispersed through a founder effect.

The cases presented in this study were initially categorized as normal PiM carriers using the IEF detection method. However, the unexpectedly low serum AAT levels led us to suspect the presence of rare deficient alleles that were undetectable by this technique. As a result, *cis* variations may be more common than previously described, as they may be missed by IEF or genotyping methods like allele-specific PCR testing. Discrepancies between serum AAT levels and the observed phenotype are a common feature of AATD and should raise suspicion of such rare alleles [2, 9] warranting *SERPINA1* gene sequencing [2]. In Lyon and Lille laboratories, IEF and quantitative AAT dosage are systematically performed as first-line tests for the biochemical screening of AATD, followed by complete Sanger sequencing in case of discrepancy (for example low AAT level with a normal IEF profile). PiZ_marseille_ variant is therefore likely not to be missed. However, this is not the case in the Bordeaux laboratory, where quantitative AAT dosage is only performed together with a dedicated PCR for the PiS and PiZ variants. It was thus possible that some patients flagged as PiZ heterozygous were in fact PiZ_marseille_ heterozygous. We therefore performed complete Sanger sequencing of 17 patients with that profile, but the PiZ_marseille_ mutation was never observed, ruling out the hypothesis of a hidden PiZ_marseille_ profile. It should be noted that the absence of the PiZ_marseille_ variant in the routine clinical practice of the Lyon, Lille, and Bordeaux reference AAT laboratories is also in itself a strong argument for a founder effect in the South East of France.

The presence of two heterozygous missense variations *in cis* on a same *SERPINA1* allele is not novel and has already been reported in various studies. Examples include associations between the Null Canada and PiZ variants [25], the PiS and PiZ alleles [26] or the PiS variant and other missense variants in cis [27]. The identification of rare *SERPINA1* variants combining the PiZ or PiS mutation with another variant on the same allele is important, as such combinations may exacerbate the functional impairment of the AAT protein and thereby influence clinical outcomes [27]. A similar scenario is observed with the *TYR* gene, in which certain variant combinations can cause albinism. The two *TYR* genotypes c.575C > A and c.1205 G > A arose on different ancestral haplotypes and each reduces pigmentation. While both variants are individually common, their co-occurrence on a recombinant haplotype (TYR c.[575C > A;1205 G > A] p.[(Ser192Tyr);(Arg402Gln)]) is relatively rare. Together, they result in an additive reduction in tyrosinase function, greater than the effect of each variant alone [28].

The rare allele detected here in combination with PiZ was the PiZ_marseille_ variant. This latter exhibits an abnormal PiZ pattern on isoelectric focusing caused by the absence of the N-terminal glycosylation site. Consequently, the PiZ_marseille_ protein migrates more extensively toward the cathode [29]. This variant, notable for its association with both liver and lung diseases and first described in 1997 by *Lovegrove et al.*, was identified in a heterozygous woman with a history of three perinatal deaths due to fulminant liver disease and no surviving offspring [30]. The PiZ_marseille_ protein is active as a protease inhibitor but appears to be deficient in plasma. One of the three N-glycosylation sites in the PiZ_marseille_ protein is disrupted by a mutation corresponding to a C to T transition (c.326C > T), changing ACG (Thr) to ATG (Met) at codon 109 (NM_000295.5:c.326C > T; p.(Thr109Met)) [30]. This amino-acid substitution disrupts the N-glycosylation site starting at Asn 107 (Asn-Leu-Thr), preventing glycosylation of the PiZ_marseille_ protein at residue 107. Additionally, and in accordance with our results (Fig. 4), *Samandari and Brown* have shown *in cellulo* that a mutant protein corresponding to the PiZ_marseille_ mutation is largely retained in the ER of hepatocytes, contributing to the low plasma concentration observed for this mutant [31]. Consequently, in addition to the liver damage described in that study, the PiZ_marseille_ variant might theoretically be associated with an increased risk of developing lung disease, although this was not observed in our cohort. Only minimal paraseptal emphysema at the apices was noted in the grandfather of the first family (Fig. 1A). Taking advantage of this study, we reviewed the clinical information of patients genotyped as PiZ_marseille_ in Lille. Three patients were identified, and none of them developed lung disease. Interestingly, *Bates et al.* [29] reported a patient with the PiZ_marseille_ mutation in compound heterozygosity with the PiZ variant. Contrary to expectations, this patient presented with breathlessness and a history of chronic fatigue, but without any evidence of liver disease or significant impairment of respiratory function [29]. Taken together, these observations suggest that significant pulmonary involvement is unlikely in these carriers, although longer follow-up may be informative.

There is considerable heterogeneity in clinical manifestations and AAT concentration levels across the different cases identified in our study, even though they share the same *SERPINA1* genotype (Supplemental Table 1). For example, the AAT level of Case 2 (0.92 g/L) seems to be significantly elevated but it was measured during an inflammatory period: hepatic synthesis factor 149% of normal and plasmatic fibrinogen at 4.55 g/L (normal range 1.80–4.0 g/L). Indeed, AAT is a serine protease inhibitor synthesized by hepatocytes in response to inflammation. Its concentration in the blood can rise by 75–100% due to inflammation, infection, or injury [32]. Apart from the inflammatory state, the observed heterogeneity in clinical manifestations suggests that additional factors, such as genetic modifiers, are likely to influence disease severity. This variability underscores the complex interplay between genotype, environment, and potential genetic modifiers, reflecting the incomplete penetrance and multifactorial nature of liver involvement, not only for the PiZ_marseille_ but also for other AATD variants, whether in the homozygous or heterozygous state [33]. In this context, our study of the Marseille family suggests that certain maternal variants, shared among the siblings, could contribute to this phenomenon, given that the father exhibits only a mild clinical phenotype, whereas the children display more severe liver involvement.

Based on their segregation within the family, we identified several candidate variants, including *AGGF1*, *VNN1*, *SUV39H2*, *CRIP1*, *DPAGT1*, and *TRIM2*. Some of these, such as *AGGF1*, *VNN1* and *SUV39H2* have been already correlated with liver disease [34–36]. Notably, Angiogenic factor with G patch and FHA domains 1 (AGGF1) regulates liver fibrosis [35] and may contribute to Metabolic dysfunction-associated steatohepatitis (MASH) pathogenesis [37] just like Vanin-1 (VNN1) and Suv39h2, two histone methyltransferases [34].

More precisely, VNN1 is a transcriptional target of Suv39h2 [34]. This histone H3K9 methyltransferase represses *VNN1* transcription in hepatocytes exposed to free fatty acids [34]. Suv39h2 knockdown in hepatocytes attenuates MASH in mice, likely by upregulating VNN1 to enhance fatty acid oxidation [38]. Epigenetic modifications are also important in the regulation of AGGF1. DNA methylation directly modulates liver fibrosis by targeting AGGF1 [35].

Additionally, *CRIP1*, *DPAGT1*, and *TRIM2*—genes involved in autophagy responses [39–41],—may act as genetic modifiers, given that Z-variant aggregates are cleared through this degradation pathway [42, 43]. Since PiZ_marseille_ retains the ability to form aggregates (Fig. 4), alterations in this pathway, potentially mediated by mutations in these genes, could partially account for the clinical presentation observed [44]. These genes are also critical for proteasome degradation and N-glycosylation, two other fundamental pathways in AAT biogenesis that are disrupted in the pathogenicity of the PiZ_marseille_ variant (Fig. 3A–C) [39, 40]. Dysregulation of these pathways may influence the severity of AATD-related clinical manifestations associated with the PiZ_marseille_ variant.

Even though our proteomic analysis was performed on a single patient, due to the extreme rarity of this genotype, these results should be considered preliminary and will require confirmation in future studies. Nevertheless, they open a novel research avenue and generate testable hypotheses. In particular, our proteomic data highlight the potential involvement of neutrophil degranulation and estrogen biosynthesis (Fig. 5). Numerous proteins were identified within the neutrophil degranulation pathway, including ACAA1, ALAD, ALDOC, ANXA2, ARSB, ASAH1, B2M, BST2, CD44, CSTB, CTSA, CTSH, CTSS, DIAPH1, DPP7, DYNLL1, FTH1, FTL, FUCA2, GALNS, GSTP1, GUSB, HBB, HEBP2, HLA-B, HRNR, HSP90AB1, IGF2R, LAMP1, LAMP2, LCN2, LGALS3, PKM, PSAP, PSMD6, RAB5C, SERPINB1, SNAP29, STBD1, TXNDC5, UBR4, VAT1, and XRCC6. Future studies involving additional patients and using multiplex immunohistochemistry or spatial proteomic imaging approaches will be essential to confirm these findings, assess their generalizability, and strengthen confidence in these results. Importantly, neutrophil elastase, a key enzyme whose natural inhibitor is AAT, plays a crucial physiological role in host defense by degrading foreign microorganisms and organic molecules that have been phagocytosed by neutrophils [45]. Neutrophil elastase also degrades various substrates, including elastin (its most critical target) [45]. The role of neutrophils in liver disease pathogenesis has garnered significant interest in recent years [46]. Since neutrophils routinely patrol liver sinusoids and since the liver contains few resident neutrophils, their excessive activation can result in liver damage [46]. However, their role in liver fibrogenesis remains a subject of debate [46]. A similar uncertainty surrounds estrogen biosynthesis. Several studies have highlighted the involvement of estrogens in chronic liver diseases [47]. The specific contributions of these two biological pathways—neutrophil degranulation and estrogen biosynthesis—to PiZ_marseille_ proteotoxicity, as well as to PiZZ proteotoxicity, warrant further investigations through additional experiments.

Although we identified a new AATD variant and provided important insights into its role in AATD-mediated liver disease, we acknowledge several limitations of this study. In particular, the impact of the maternal variants shared by the three affected children remains unclear, as does their potential contribution to the distinct pathways highlighted by the proteomic analysis. These proteomic results will also need to be reproduced in additional patients to confirm their significance and generalizability. Considering the REVEL or Splice-AI scores, the *HGD* or *DGAGT1* variants could be the most promising. Consequently, based on these findings, further examination is needed to both characterize the impact of each highlighted mutation on its encoded protein and to better understand and clarify the contributions of these genes to disease progression. Nevertheless, a STRING analysis revealed connections between some candidate genes identified through exome sequencing and the proteins comprising the most enriched pathway (neutrophil degranulation) for the PiMZ_marseille_ genotype (Supplemental Figure S2).

In summary, this study provides novel insights into the genetic basis of AATD and underscores the clinical importance of identifying rare variants of the AAT protein. Our data suggest that the genetic modifications underlying PiZ_marseille_-associated liver disease likely involve variations in multiple genes rather than a single one. By providing preliminary clues about potential molecular mechanisms driving AATD-associated liver disease, our findings could lay the groundwork for personalized approaches to diagnosis, prognosis, and therapeutic intervention in affected patients. For the next steps, functional studies of genetic modifiers, longitudinal follow-up of carriers, and testing of therapeutic responses are all highly relevant and will need to be addressed in future studies.

## Electronic supplementary material

Below is the link to the electronic supplementary material.

Supplementary Material 1

## Data Availability

The data supporting the findings of this study are available through the ProteomeXchange Consortium via PRIDE, and from the corresponding author upon reasonable request.
