# Supplementary material for: Characterization of a novel SERPINA1 variant carrying two missense mutations: molecular mechanisms and functional impact
Source: Orphanet J Rare Dis. 2025 Nov 21;20:615. doi: 10.1186/s13023-025-04142-z (PMC12659552; doi:10.1186/s13023-025-04142-z)

Characterization of a novel *SERPINA1* variant carrying two missense mutations: molecular mechanisms and functional impact

Short title: $PiZ_{marseille}$, a new variant of *SERPINA1* Gene Causing Alpha-1 Antitrypsin Deficiency.

Celine Leon[1#], Marie-Françoise Odou[2#], Bertrand Roquelaure[3#], Louis Lebreton[4], Mathias Ruiz[5], Carolin Victoria Schneider[6], Celine Renoux[7], Aurélie Evrard[2], Malika Balduyck[2], Magali Dechomet[13], Christine Lombard[13], Mathilde Butori-Pepino[14], Kai Markus Schneider[6], Victor Marin[4], Sylvaine di-Tomasso[8], Cyril Dourthe[1,8], Jean-William Dupuy[8], Anne-Aurélie Raymond[1,8], Sophie Collardeau-Frachon[9], Aurélie Haffner[10], Radia Fritih[10], Emmanuelle Goubert[3], Vanna Geromel[11], Philippe Joly[7], Alexandre Fabre[3,12], Marion Bouchecareilh[1&].

1. University of Bordeaux, CNRS, INSERM, BRIC, U1312, Bordeaux, France

2. CHU Lille, Department of Biochemistry and Molecular Biology 'Hormonologie, Métabolisme-Nutrition, Oncologie', Lille, France,

3. Department of Pediatrics, Hôpital de la Timone Enfants, Assistance-Publique des Hôpitaux de Marseille (AP-HM), Marseille, France.

4. Département de Biochimie, Hôpital Pellegrin, Centre Hospitalier Universitaire de Bordeaux, Bordeaux, France

5. Department of Pediatric Hepatology, Gastroenterology and Nutrition, French Reference Center for Biliary Atresia and Genetic Cholestasis, European Reference Network Rare-Liver, Hôpital Femme-Mère-Enfant, Hospices Civils de Lyon, France.

6. Department of Gastroenterology, Metabolic Diseases and Intensive Care, University Hospital RWTH Aachen, Aachen, Germany.

7. Service de Biochimie et de Biologie Moléculaire, Centre de Biologie et de Pathologie Est, Hospices Civils de Lyon, Bron, France.

8. Univ. Bordeaux, CNRS, INSERM, TBM-Core, US5, UAR 3427, OncoProt, F-33000 Bordeaux, France

9. Department of Pathology, Hôpital Femme Mère Enfant, Hospices Civils de Lyon, Lyon, France.

10. Department of Pathology, Hôpital de la Timone, Assistance-Publique des Hôpitaux de Marseille (AP-HM), Marseille, France.

11. Service de génétique-oncogénétique moléculaire Eurofins-Biomnis, Lyon, France.

12. Aix Marseille University, MMG, INSERM, Marseille, France.

13. Department of Biological Immunology, Hôpital Lyon Sud, Hospices Civils de Lyon, France

14. Pediatric Gastroenterology Department, Hôpitaux pédiatriques de Nice CHU-Lenval, Nice, France.

15. Univ. Lille, Inserm, CHU Lille, U1286 – Infinite –, F-59000 Lille, France

16. EA 7364 RADEME, laboratoire de biochimie et biologie moléculaire, Université de Lille, France

# Contributed equally to this work with: Celine Leon, Marie-Françoise Odou, Bertrand Roquelaure

& Corresponding author: marion.bouchecareilh@cnrs.fr

Table of contents

Supplementary Figure 1 3

Supplementary Figure 2 4

Supplementary Table 1 5

Supplementary Table 2 6

Supplementary Table 3 7

Supplementary Table 4 8

Supplementary Table 5 9

Supplementary Table 6 10

Full size images of the main figure 4 11

Supplemental Figure 1: Evolution of liver function and AAT plasma levels in patient case 1

ASAT: Aspartate aminotransferase ; ALAT: Alanine aminotransferase ; PAL: Alkaline phosphatase ; GGT: Gamma-glutamyl transferase.

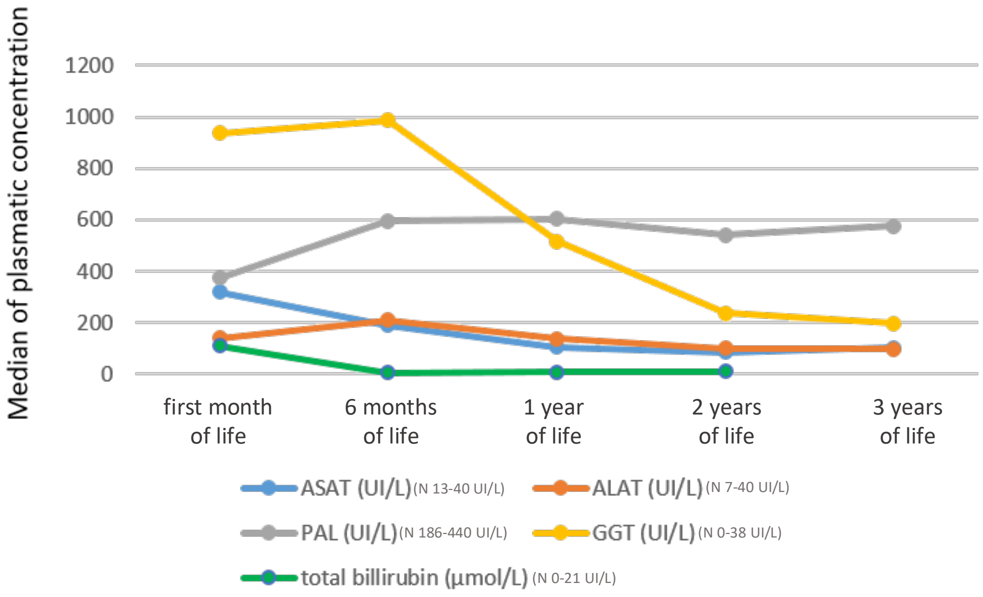

Supplemental Figure 2: String software analysis

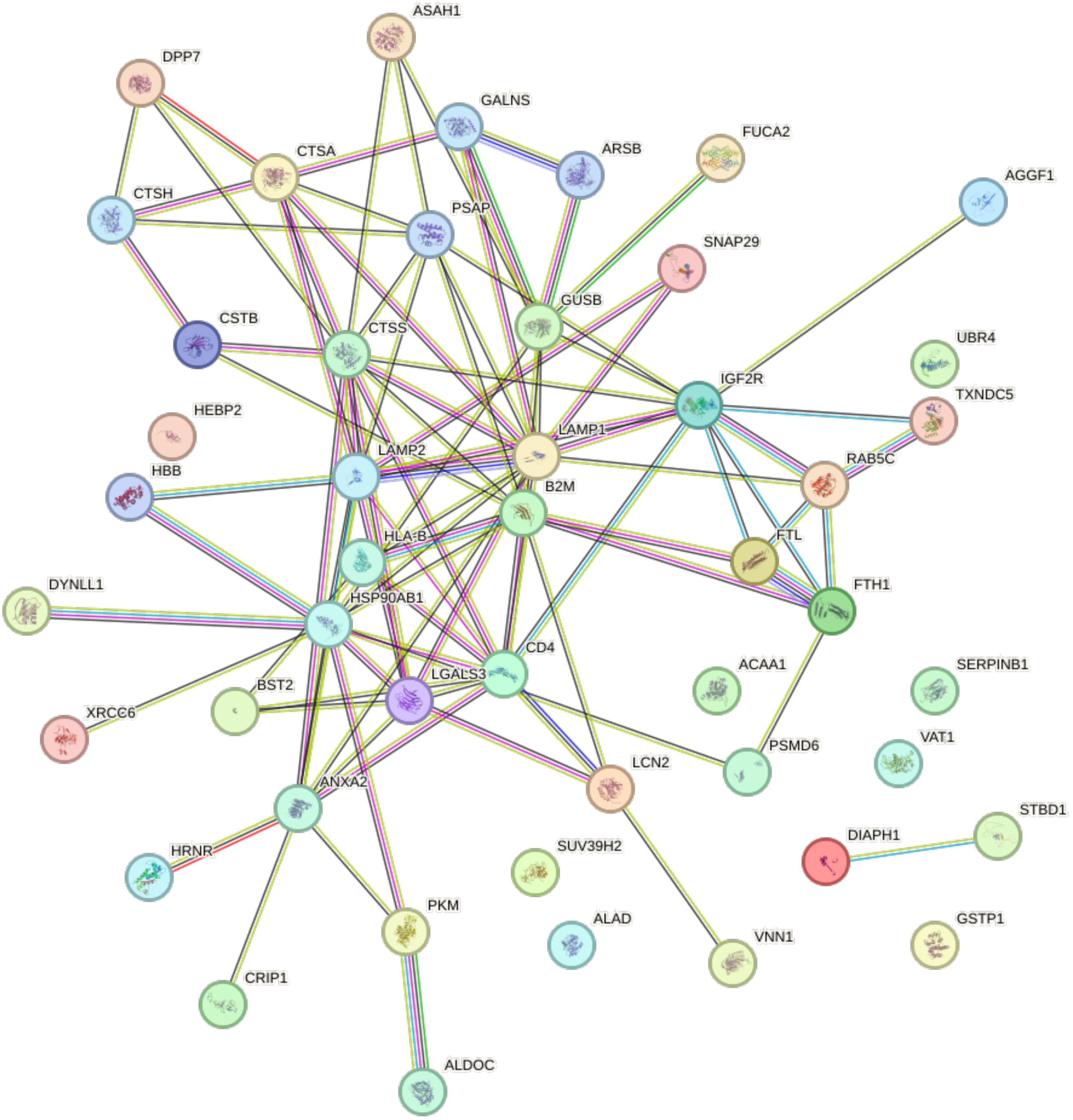

Supplemental Table 1: Median of all biological results of liver function

ASAT: Aspartate aminotransferase ; ALAT: Alanine aminotransferase ; PAL: Alkaline phosphatase ; GGT: Gamma-glutamyl transferase ; AAT : Alpha 1-Antitrypsin.

	**ASAT** (UI/L) (N 13-40)	**ALAT** (UI/L) (N 7-40)	**PAL** (UI/L) (N 186-440)	**GGT**(UI/L) (N 0-38)	**Total billirubin** (µmol/L) (N 0-21)	**AAT** (g/L) (N 0.9-2)
**Case 1**	114	138	550	715	12	0.85
**Case 2**	120	58	680	240	17	0.92
**Case 3**	117.5	162	574	336.5	[redacted]	0.81
**Case 4**	40	58	85	153	6	0.62
**Case 5**	24	34	77	39	[redacted]	0.60

Supplemental Table 2: Additional examination for cholestatic or metabolic diseases

Analysis	Details
Metabolic Investigation	Ammonia : 60 µmol/L
	Pyruvate : 83.94
	Lactate : 1.40 mmol/L
	Plasmatic Amino Acid Chromatography : decrease in citrulline (11µmol/L), tyrosine (46µmol/L) and phenylalanine(40µmol/L), without suggesting an aminoacidopathy.
	Urinary Amino Acid Chromatography : The chromatigraphic profile of the sample analysed showed a non-specific generalised hyperaminoaciduria, a priori linked to the absence of fasting.
	Urinary organic acid chromatography : Negative
	Acyl Carnitine Profile : normal
	Galactosemia : negative
	Nieman Pick C : oxysterol: normal levels inconsistent with the lyosphingolipid profile<br>Cholitriosidase: no increase<br>Lyosphingolipids: elevation of lyoSM509/N-Palmitoyl-Phosphcholineserine. No increase in oxysterols: discordant profile probably related to hypertriglyceridaemia.
Infectious and Immune Assessment	Serologies for VZV, CMV, EBV, Parvovirus, Rubella, Toxo indicating maternal immunity;<br>negative results for VHA, VHB, VHC, VHE, HSV, and Syphilis
PCR Results	Undetectable EBV, initially negative CMV, negative HSV, and negative HVA and VHE
Other	Normal quantification assay for immunoglobulin and Lymphocyte Typing
Cortisol	178nmol/l
Immunoreactive trypsin (guthrie test)	10.2
Sweat test	13mmol/l
Stool elastase	330µg/g
Lipid profile	Total cholesterol : 8,28 mmol/l (N < 6,20),<br>LDL (low density lipoprotein) cholesterol : 6,33 mmol/l (N < 4,92)<br>HDL (low density lipoprotein)cholesterol : 0,69 mmol/L (N > 1,45)<br>Triglycerides : 2,78 mmol/l (N < 1,7)
Cardiac echography	Normal
Ophthalmological test	Normal
Abdomino-pelvic echography	Hyperechogenicity of the periportal spaces
Chest radiograph	Normal

Supplemental Table 3: UK Biobank Analysis

$Pi_{bristol}$ (rs199422213_A)

PIS	PiZ			Frequency	Percent	Valid Percent	Cumulative Percent
.	.	Missing	System	14241	100,0		
	0	Valid	0	314	99,7	100,0	100,0
		Missing	System	1	0,3		
		Total		315	100,0		
	1	Valid	0	12	100,0	100,0	100,0
0	.	Valid	0	402	100,0	100,0	100,0
	0	Valid	0	426685	99,9	100,0	100,0
			1	46	0,0	0,0	100,0
			Total	426731	100,0	100,0	
		Missing	System	175	0,0		
		Total		426906	100,0		
	1	Valid	0	17164	99,9	100,0	100,0
		Missing	System	10	0,1		
		Total		17174	100,0		
	2	Valid	0	141	100,0	100,0	100,0
1	.	Valid	0	37	100,0	100,0	100,0
	0	Valid	0	41268	99,9	100,0	100,0
			1	3	0,0	0,0	100,0
			Total	41271	100,0	100,0	
		Missing	System	20	0,0		
		Total		41291	100,0		
	1	Valid	0	874	100,0	100,0	100,0
2	0	Valid	0	1018	100,0	100,0	100,0

Supplemental Table 4: Genes of the panel involved in genetic cholestasis or jaundice

ABCB11 (NM_003742), ABCB4 (NM_000443), ABCC2 (NM_000392), ABCC12 (NM_033226), ABCG5 (NM_022436) , ABCG8 (NM_022437), ABCD3 (NM_002858), ACOX2 (NM_003500), ADK (NM_006721), AGL (NM_000028), AIRE (NM_000383), AKR1D1 (NM_005989), ALDOB (NM_000035), AMACR (NM_014324), AQP8 (NM_001169), ATP7A (NM_000052), ATP7B (NM_000053), ATP8B1 (NM_005603), BAAT (NM_001701), CCBE1 (NM_133459), CFTR (NM_000492), CIRH1A (NM_032830), CLDN1 (NM_021101), CLDN6 (NM_021195), CLDN9 (NM_020982), CP (NM_000096), CPT1A (NM_001876), CTC1 (NM_025099), CYP27A1 (NM_000784), CYP7A1 (NM_000780), CYP7B1 (NM_004820), DCDC2 (NM_016356), DGUOK (NM_080916), DKC1 (NM_001363), DLD (NM_000108), FAH (NM_000137), FBP1 (NM_000507), FOPV (C4orf54)(NM_001354435), FOXF (NM_001451), FUT2 (NM_000511), GALE (NM_000403), GALT (NM_000155), GATA6 (NM_005257), GBE1 (NM_000158), GFM1 (NM_024996), GPBAR1 (NM_001077191), GYG2 (NM_003918), GYS2 (NM_021957), HAMP (NM_021175), HFE (NM_000410), HFN1A (NM_000545), HFN1B (NM_000458), HSD17B4 (NM_000414), HSD3B7 (NM_025193), IARS (NM_002161), IFT140 (NM_014714), IFT172 (NM_015662), IL18BP (NM_173042), JAG1 (NM_000214), KCNN3 (NM_002249), KIF12 (NM_138424), LARS (NM_020117), LFNG (NM_002304), LSR (NM_015925), MARS (NM_004990), MFNG (NM_002405), MPV17 (NM_002437), MTOR (NM_004958), MYO5B (NM_001080467), NBAS (NM_015909), NFAM1 (NM_145912), NOTCH1 (NM_017617), NOTCH2 (NM_024408), NR1H4 (NM_00512), PHKA2 (NM_000292), PHKB (NM_000293, NM_001031835), PHKG2 (NM_000294), PKD1L1 (NM_138295), PKHD1 (NM_138694), PLECTIN (NM_000445), PNPLA3 (NM_025225), POGLUT1 (NM_152305), POLG (NM_002693), PPM1F (NM_014634), PPP1R15B (NM_032833), PRKCSH (NM_001001329), PYGL (NM_002863), RAB11A (NM_004663), RASA1 (NM_002890), RASAL3 (NM_022904), RDX (NM_002906), RFNG (NM_002917), RINT1 (NM_021930), RRAGC (NM_022157), RTEL1 (NM_032957), SCO1 (NM_004589), SCYL1 (NM_020680), SEC63 (NM_007214), SERPINA1 (NM_000295), SI (NM_001041), SLC10A1 (NM_003049), SLC10A2 (NM_000452), SLC11A2 (NM_00617), SLC25A13 (NM_014251), SLC27A5 (NM_012254), SLC2A2 (NM_000340), SLC2A5 (NM_003039), SLC4A2 (NM_003040), SLC40A1 (NM_014585), SLC5A1 (NM_000343), SLCO1B1 (NM_006446), SLCO1B3 (NM_019844), TALDO1 (NM_006755), TEK (NM_000459), TERT (NM_198253), THBS2 (NM_003247), TKFC (NM_015533), TJP2 (NM_004817), TFR2 (NM_003321), TRMU (NM_018006), TTC37 (NM_014639), TUFM (NM_003321), UGT1A1 (NM_000463), UNC45A (NM_018671), USP53 (NM_019050), VIL1 (NM_007127), VIPAS39 (NM_022067), VPS33B (NM_018668), WDR83OS (c19orf56) (NM_016145), ZFYVE19 (NM_032850).

Supplemental Table 5: Rare gene variants inherited from mother and shared by cases 1, 2, 3.

Gene	Transcrit Mane Select	HGVS DNA variant	HGVS protein variant	GnomAD v4 Exome frequency	REVEL*	SPLICE AI$
TTN	NM_001267550	c.79903G>A	p.(Asp26635Asn)	NA	0.117	-
FYB	NM_001465	c.-27-5876G>T	p.(?)	NA	-	0
AGGF1	NM_018046	c.516+4A>G	p.(?)	NA	-	0.96
ADGRF4	NM_153838	c.1089del	p.(Cys364AlafsTer3)	NA	-	-
VNN1	NM_004666	c.956A>G	p.(Tyr319Cys)	NA	0.633	-
BAZ1B	NM_032408	c.1883C>G	p.(Ala628Gly)	NA	0.079	-
SUV39H2	NM_001193424	c.1123_1126+1del	p.(?)	NA	-	NA
CRIP1	NM_001311	c.40+6C>T	p.(?)	NA	-	0
HGD	NM_000187	c.767C>A	p.(Ala256Asp)	1.368e-06	0.846	-
DPAGT1	NM_001382	c.626A>C	p.(Asn209Thr)	6.84e-07	0.889	-
TRAK1	NM_001042646	c.286+23941C>T	p.(?)	2.385e-05		-
TRIM2	NM_015271.5	c.1043C>G	p.(Thr348Ser)	1.574e-05	0.219	-
ALKBH8	NM_138775	c.997T>C	p.(Phe333Leu)	2.226e-05	0.281	-
RALGAPA1	NM_001346249	c.2861A>G	p.(His954Arg)	3.304e-05	NA	-
NBEAL2	NM_015175	c.2459C>T	p.(Thr820Ile)	2.177e-05	0.078	-
CYP26B1	NM_019885	c.705+7A>G	p.(?)	0.0015	-	0
TAAR2	NM_001033080	c.143C>A	p.(Ser48Ter)	2.736e-05	-	-
TENM2	NM_001395460	c.7633A>G	p.(Ile2545Val)	0.0012	0.144	

NA : not available

*For the REVEL score, a variant is considered benign if the score is below 0.2, uncertain if the score is between 0.2 and 0.5, and damaging if the score exceeds 0.5.

$A splice variant is considered to potentially affect splicing if the Splice-AI score is above 0.8.

Supplemental Table 6: Patient's clinical history

Patient	Sex	Genotype	Type of surgery
1	Male	PiZZ	Cirrhosis/hepatectomy
2	Female	PiMZ	Biopsies
3	Male	$PiMZ_{bristol}$	Biopsies
4	Male	$PiMZ_{marseille}$	Biopsies

Full size images of the main figure 4

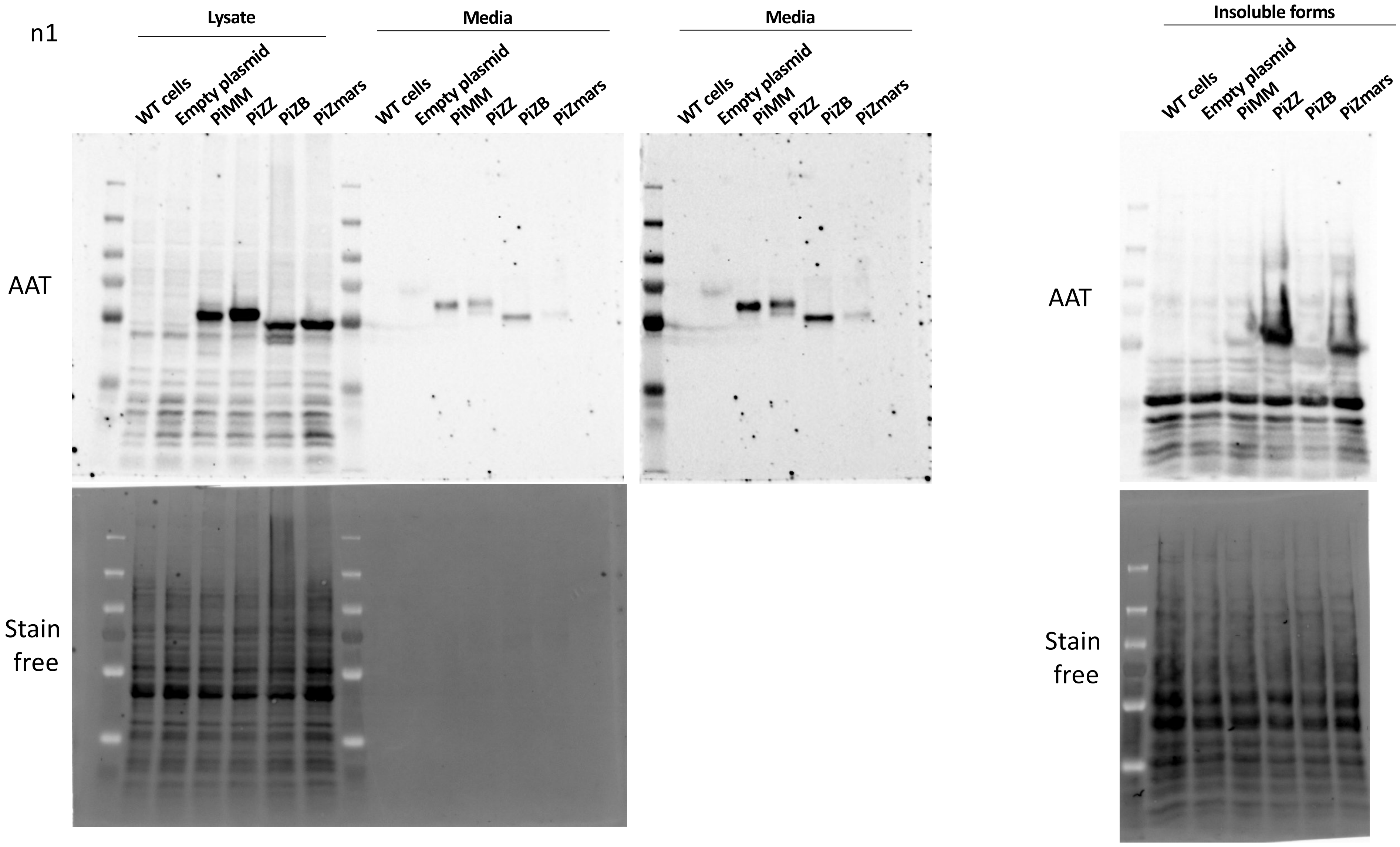
n1
Lysate
Media
Media
Insoluble forms
WT cells
Empty plasmid
PiMM
PiZZ
PiZB
PiZmars
AAT
Stain free

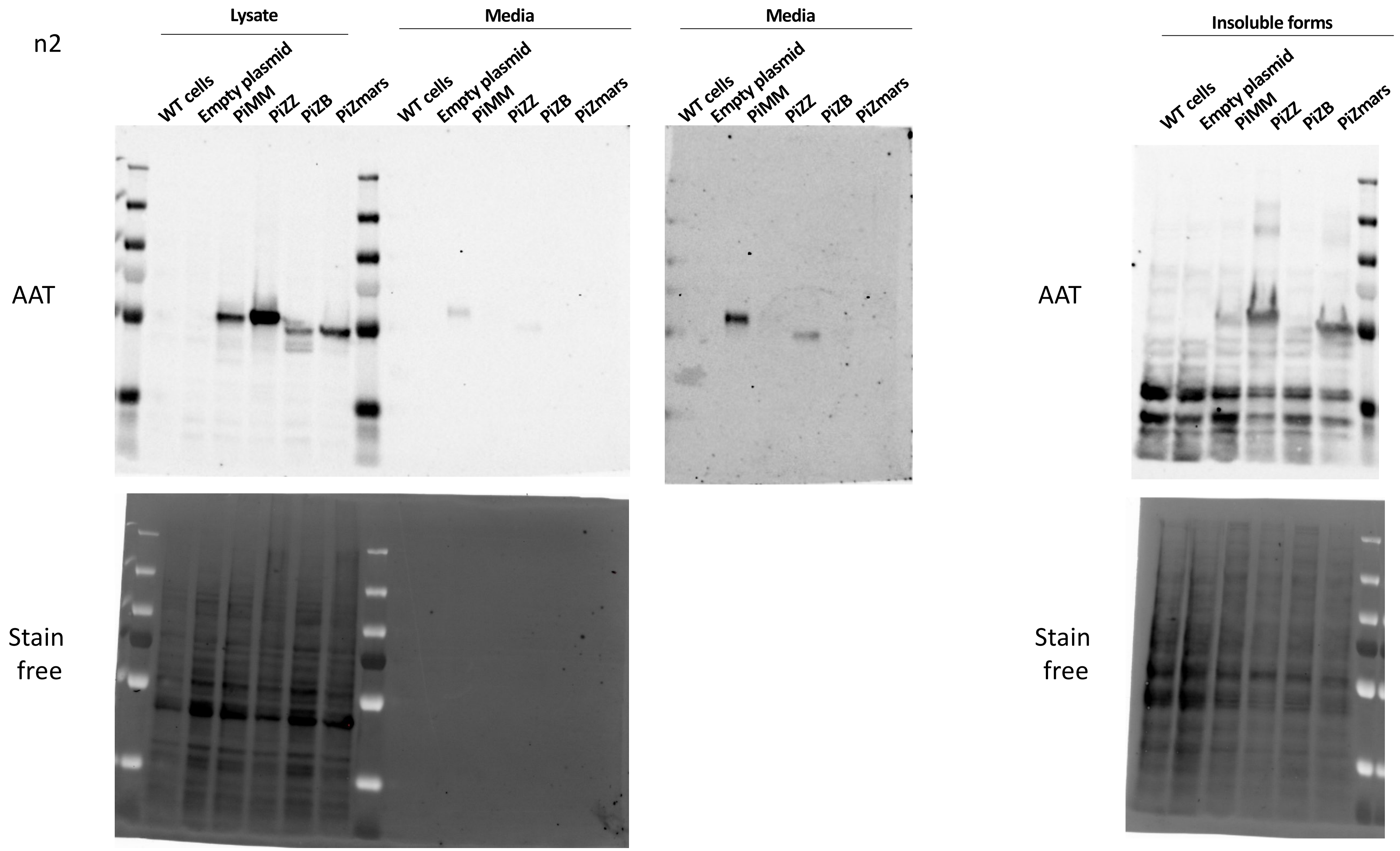
n2
Lysate
Media
Media
Insoluble forms
WT cells
Empty plasmid
PiMM
PiZZ
PiZB
PiZmars
AAT
Stain free

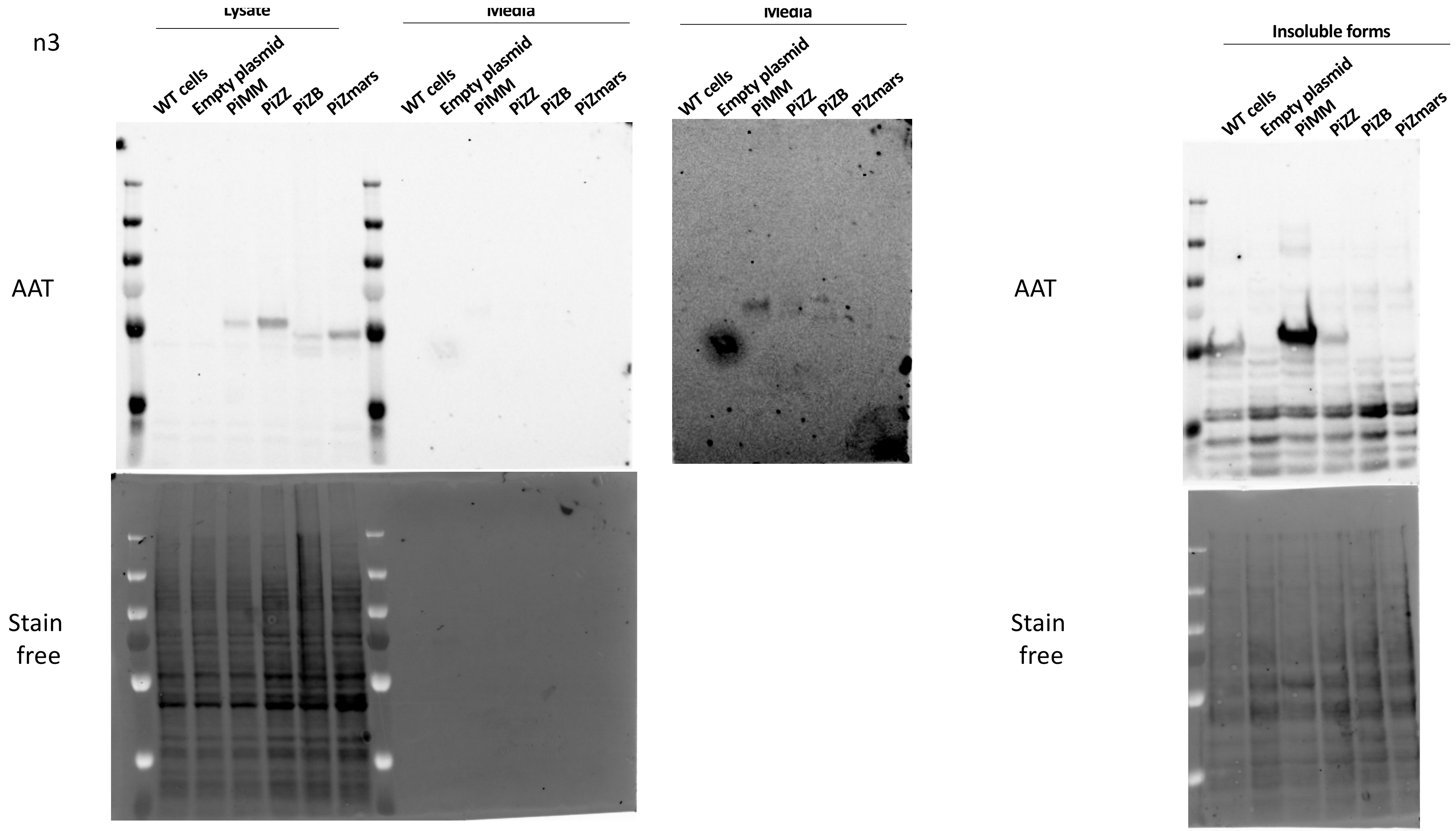
n3
Lysate
WT cells
Empty plasmid
PiMM
PiZZ
PiZB
PiZmars
Media
WT cells
Empty plasmid
PiMM
PiZZ
PiZB
PiZmars
Media
WT cells
Empty plasmid
PiMM
PiZZ
PiZB
PiZmars
Insoluble forms
WT cells
Empty plasmid
PiMM
PiZZ
PiZB
PiZmars
AAT
Stain free
AAT
Stain free

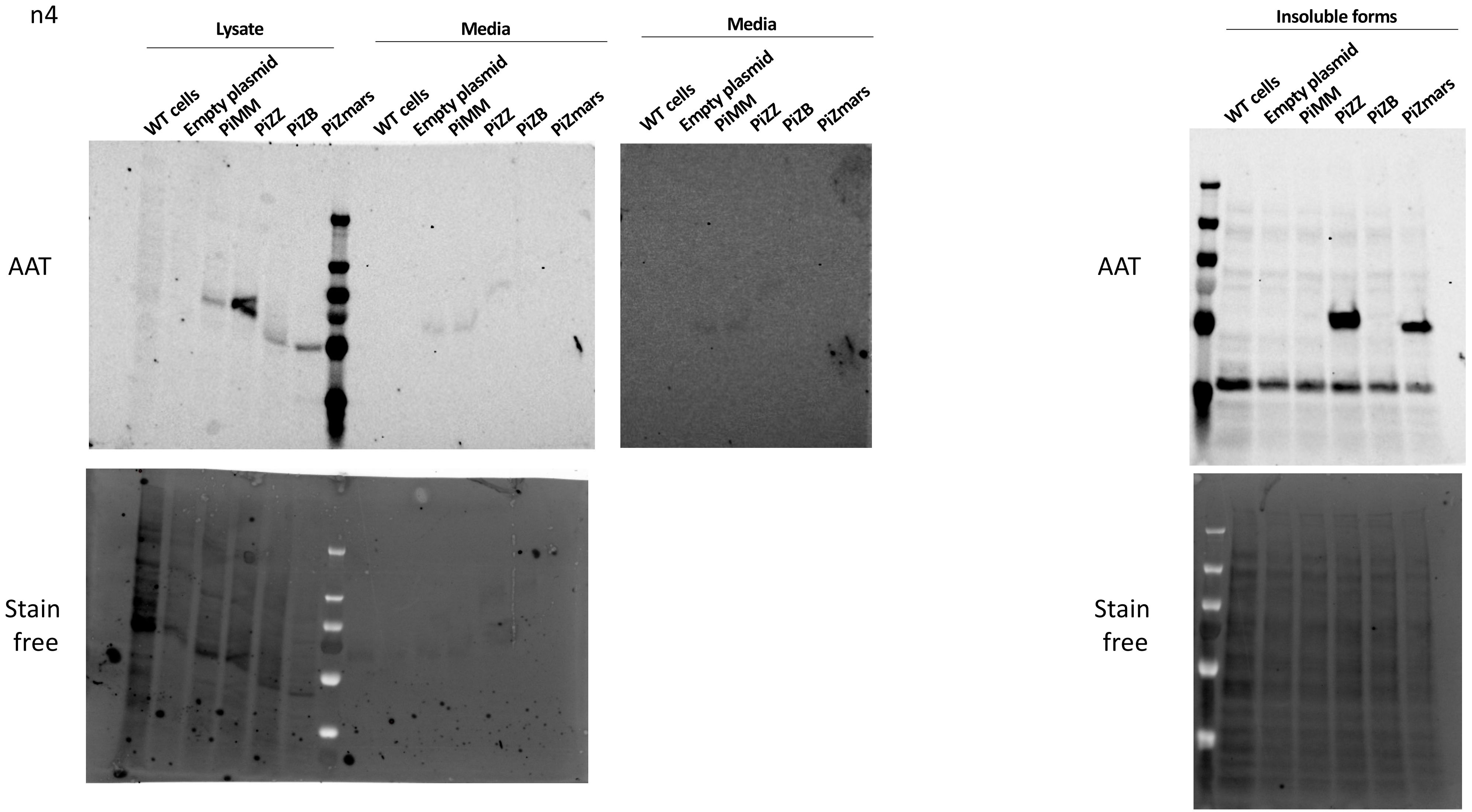
n4
Lysate
Media
Media
Insoluble forms
WT cells
Empty plasmid
PiMM
PiZZ
PiZB
PiZmars
AAT
Stain free

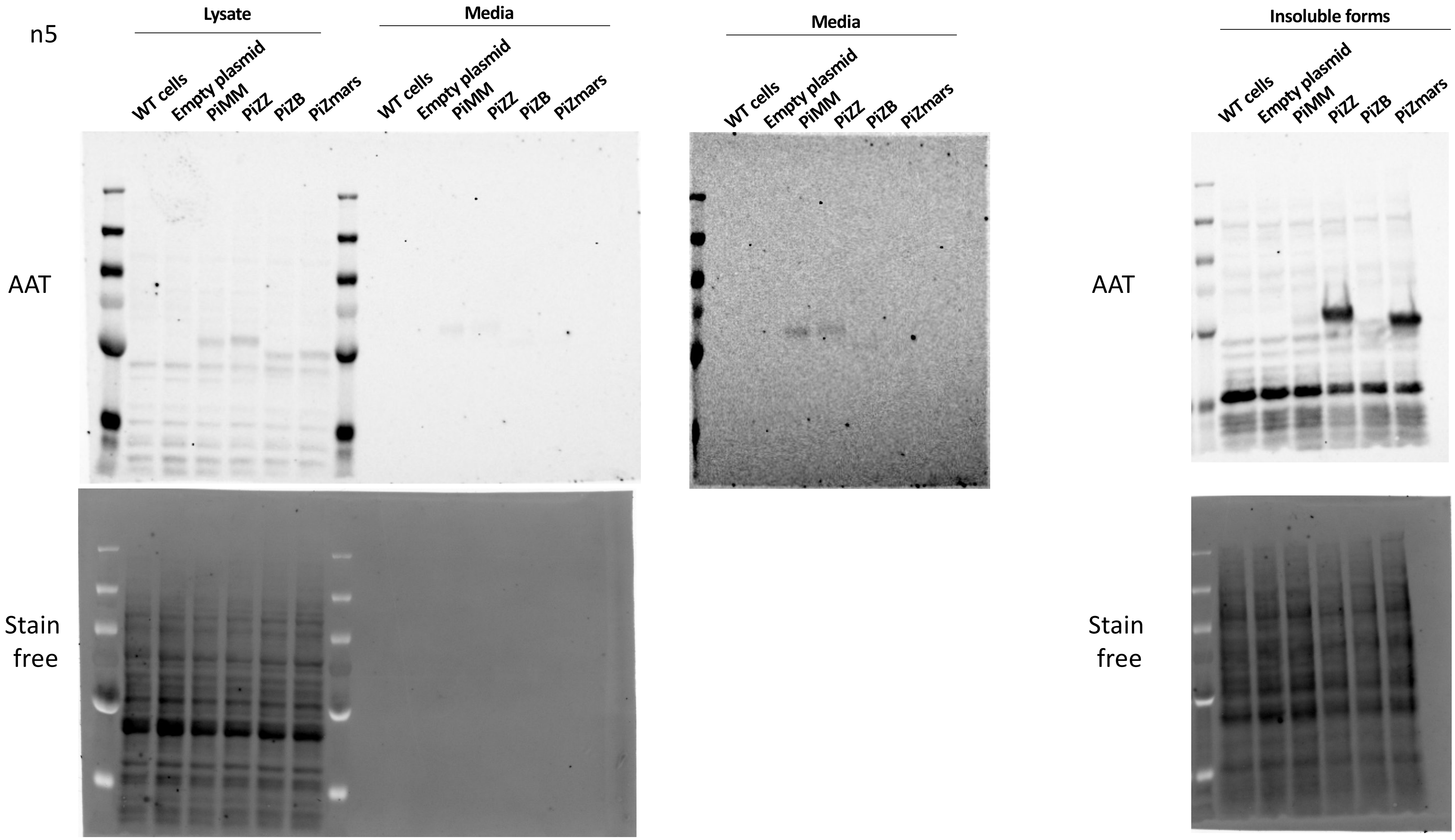

n5
Lysate
Media
Media
Insoluble forms
WT cells
Empty plasmid
PiMM
PiZZ
PiZB
PiZmars
AAT
Stain free

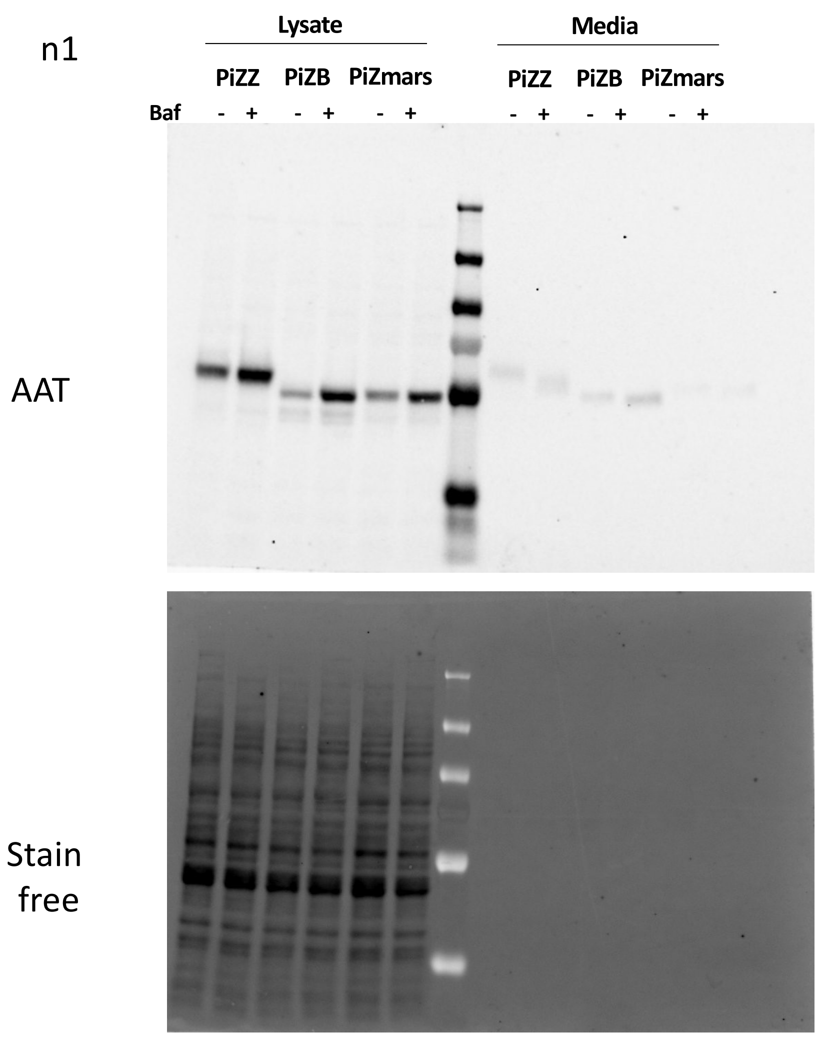
n1
Lysate
Media
PiZZ
PiZB
PiZmars
PiZZ
PiZB
PiZmars
Baf
- + - + - + - + - + - +
AAT
Stain free

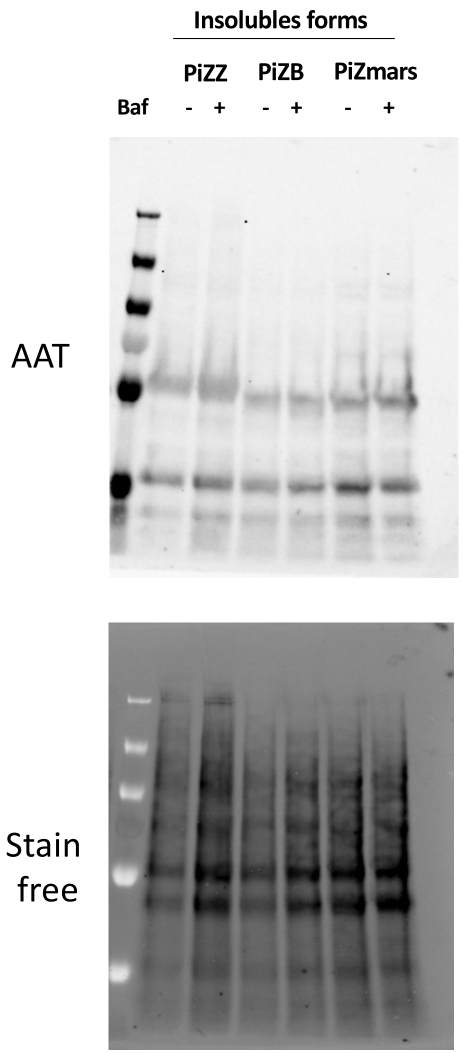
Insolubles forms
PiZZ
PiZB
PiZmars
Baf
- + - + - +
AAT
Stain free

n2

AAT

Stain free

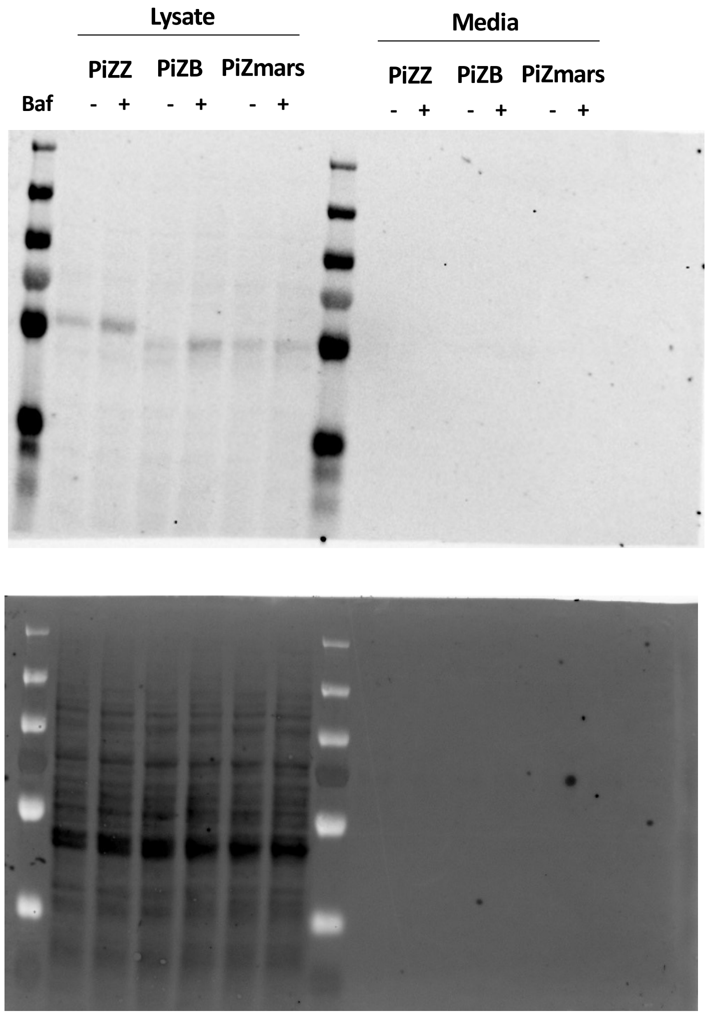

AAT

Stain free

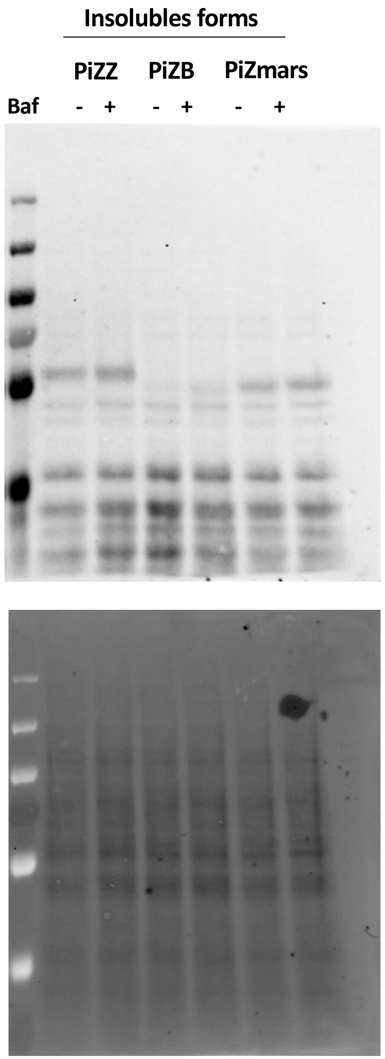

n3

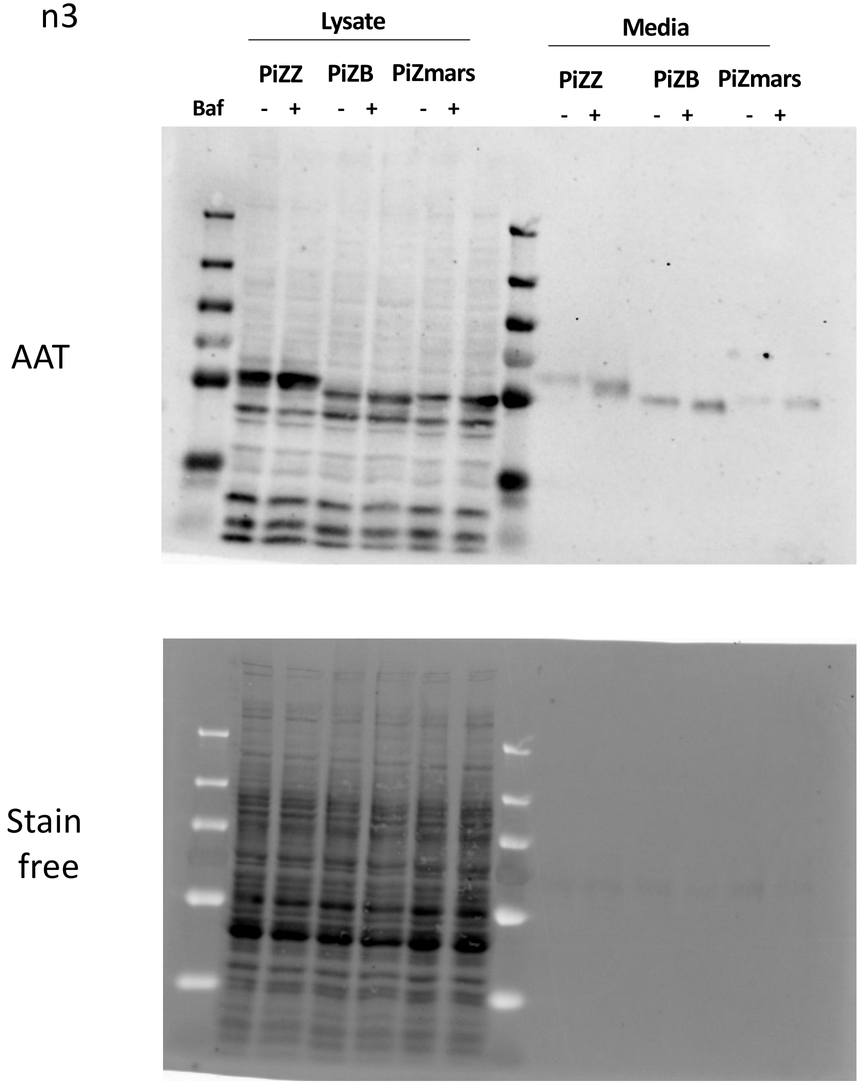

Lysate
Media
PiZZ
PiZB
PiZmars
PiZZ
PiZB
PiZmars
Baf
- +
- +
- +
- +
- +
- +
AAT
Stain free

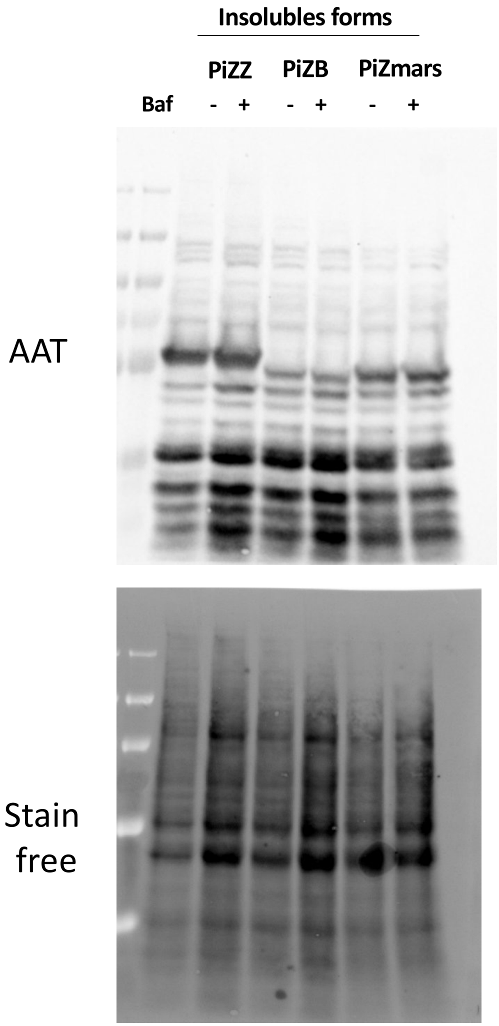

Insolubles forms
PiZZ
PiZB
PiZmars
Baf
- +
- +
- +
AAT
Stain free

n1

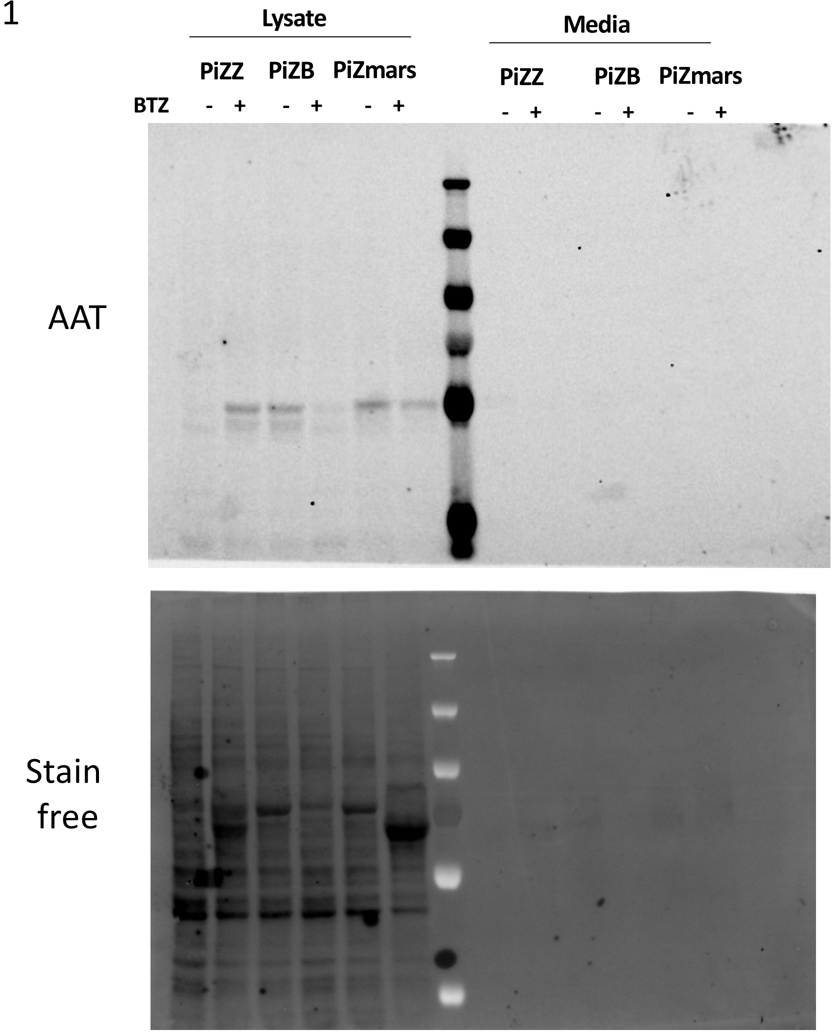
Lysate
Media
PiZZ
PiZB
PiZmars
PiZZ
PiZB
PiZmars
BTZ
- + - + - + - + - + - +
AAT
Stain free

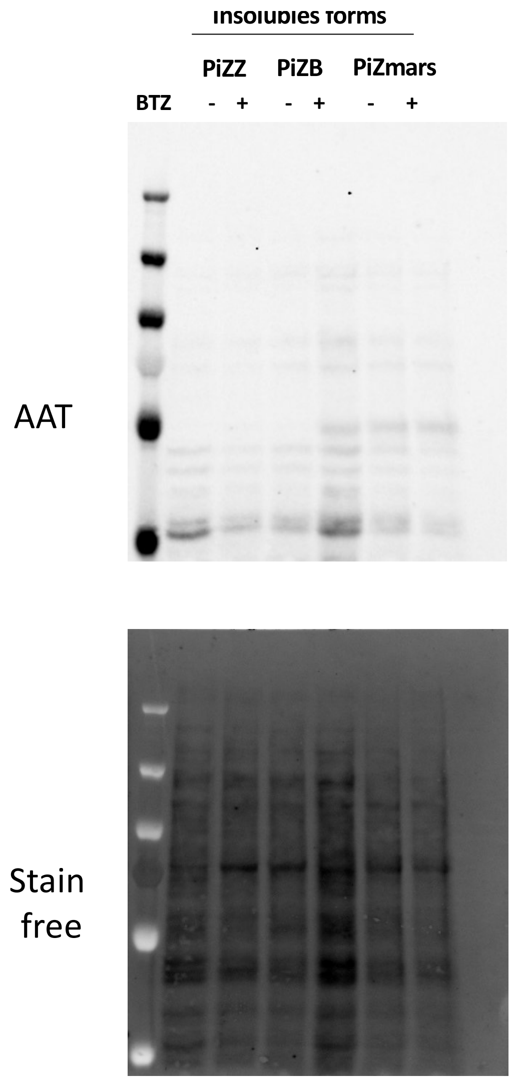
Insolubles forms
PiZZ
PiZB
PiZmars
BTZ
- + - + - +
AAT
Stain free

n2

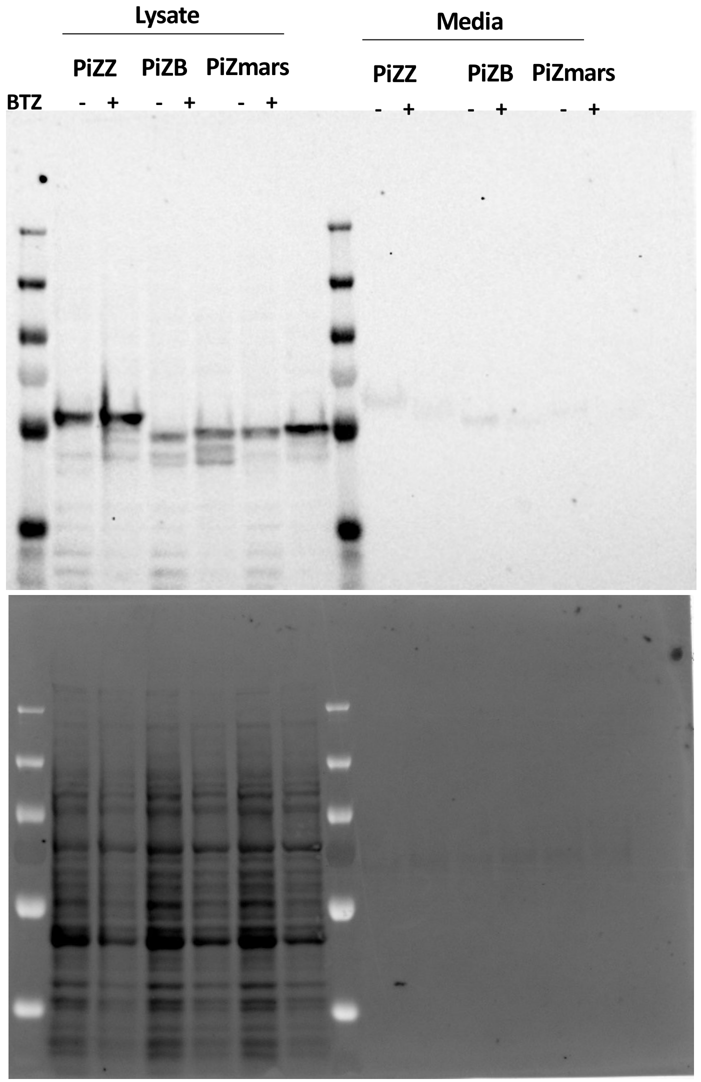
Lysate
Media
PiZZ
PiZB
PiZmars
PiZZ
PiZB
PiZmars
BTZ
- + - + - +
- + - + - +
AAT
Stain free

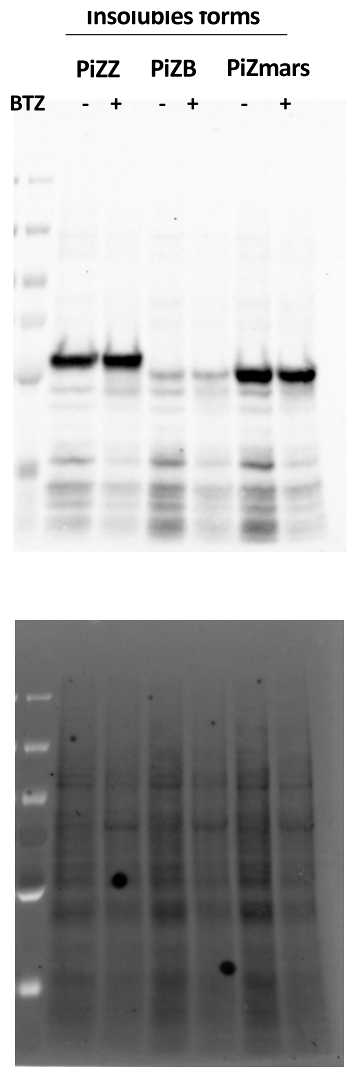
Insolubles forms
PiZZ
PiZB
PiZmars
BTZ
- + - + - +
AAT
Stain free

n3

Lysate — PiZZ, PiZB, PiZmars
Media — PiZZ, PiZB, PiZmars

BTZ - + - + - + - + - + - +

AAT

Stain free

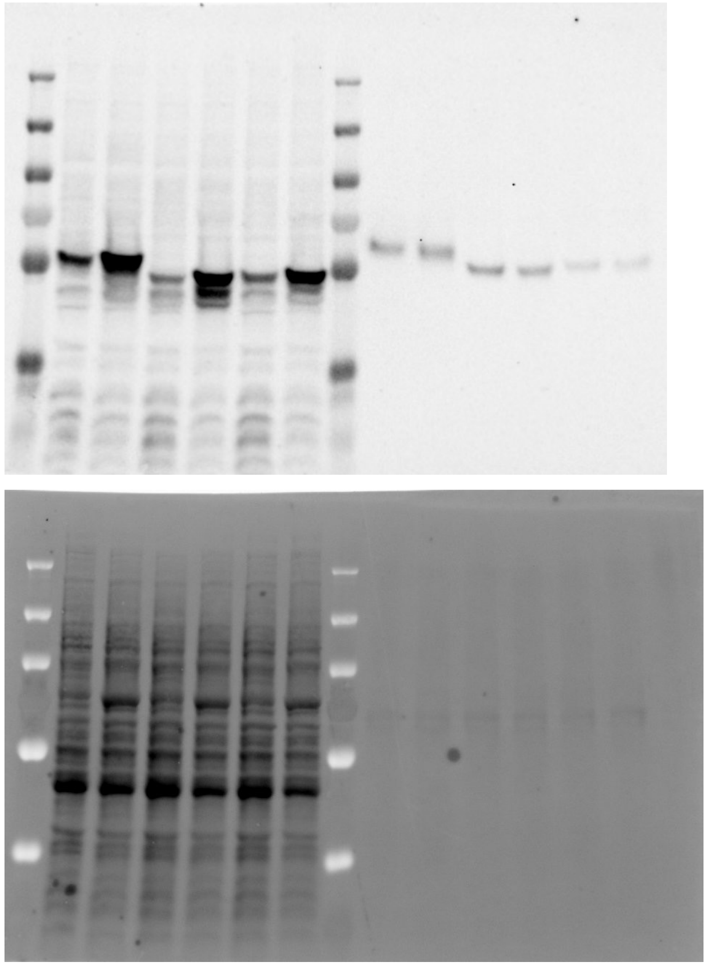

Insolubles forms — PiZZ, PiZB, PiZmars

BTZ - + - + - +

AAT

Stain free

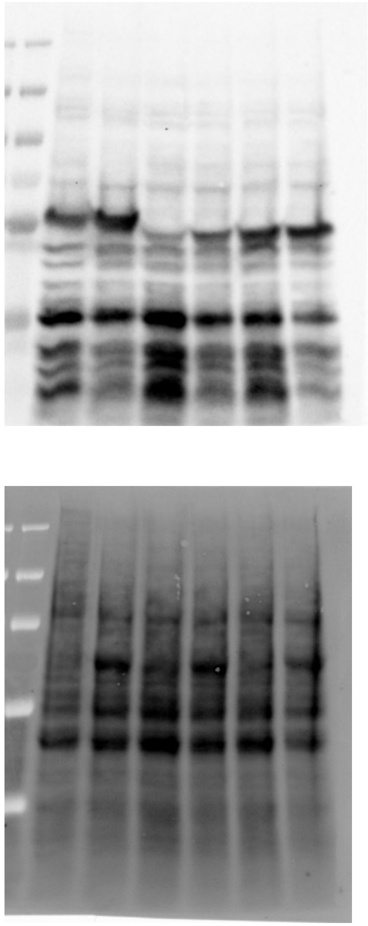

Supplement: Supplementary file 1 — Supplementary Material 1 [file 13023_2025_4142_MOESM1_ESM.pdf]
